# Identification of necroptosis-associated mRNA biomarkers in kidney clear cell carcinoma

**DOI:** 10.3389/fimmu.2025.1545486

**Published:** 2025-09-03

**Authors:** Xiaobo Zhang, Qiaoying Jin, Gafang Cheng, Haiyu Niu, Suping Yang, Shicheng Chen

**Affiliations:** ^1^ Cuiying Biomedical Research Center, The Second Hospital & Clinical Medicine School, Lanzhou University, Lanzhou, China; ^2^ Department of Oncology, The Second Hospital & Clinical Medical School, Lanzhou University, Lanzhou, China; ^3^ Department of Bioengineering and Biotechnology, College of Chemical Engineering, Huaqiao University, Xiamen, China; ^4^ Medical Laboratory Sciences Program, College of Health and Human Sciences, Northern Illinois University, DeKalb, IL, United States

**Keywords:** KIRC, necroptosis, biomarkers, prognostic, immune microenvironment, therapeutic target

## Abstract

**Introduction:**

Kidney clear cell carcinoma (KIRC) is the most common subtype of renal malignancy with a high mortality rate. It is difficult to treat and often leads to death due to its genomic heterogeneity, metastatic nature, and limited effectiveness of targeted and immunotherapies. Recent studies showed that the progression of KIRC is frequently accompanied by significant changes in necroptosis while these studies were limited by small gene sets, which increases the risk of missing low-expressed yet important genes.

**Methods:**

This study focused on necroptosis-associated genes within the context of KIRC and performed a complete closed-loop studies by gene screening, gene expression analysis, model validation and experimental translation.

**Results:**

Among screened nine core biomarkers (*RIPK1, RIPK3, MLKL*, *CASP8*, *ZBP1*, *TLR3*, *PYGL*, *TRPM7*, *PGAM5*), *CASP8* and *TRPM7* were identified as new potential biomarkers. The predictive performance of risk prognostic model for 5-year survival (AUC: 0.77 and 0.89 in training and independent/external validation cohort) outperformed prior studies by 5.5% and 17.1%, respectively. A more pronounced immune response was found with high-risk cohort, underscoring the immunosuppressive properties of tumor immune microenvironments, which evidenced by increased immune cell infiltration and elevated immunogenicity. Drug sensitivity analysis revealed that doxorubicin could serve as a promising therapeutic agent for KIRC. Furthermore, BFTC909 and CAL54 were identified as the most suitable cell lines for *in vitro* experimental translation, and highlighting three functionally significant target genes (*CASP8*, *PGAM5*, and *CPT2*).

**Conclusion:**

This study offers multi-dimensional data that support novel mechanistic investigations and provide valuable insights for developing precision immunotherapy strategies in KIRC.

## Introduction

Renal cell carcinomas (RCC) are a diverse group of cancers originating from renal tubular epithelial cells, accounting for approximately 2% of all cancer diagnoses (1–3). Among these, kidney clear cell carcinoma (KIRC) is the predominant subtype, representing about 80% of RCC cases (2, 4). Studies have showed that KIRC typically arises from mutations in the *VHL* gene located on the short arm of chromosome 3 (3p), leading to VHL protein inactivation (5, 6). This inactivation results in the overexpression of hypoxia-inducible factors (HIF-1 and HIF-2) or epigenetic silencing through promoter methylation (6, 7). Additional mutational silencing of other tumor suppressor genes on chromosome 3p may contribute to KIRC pathogenesis (8, 9). However, understanding the pathogenesis of KIRC remains limited due to its complex progression. Therefore, it is crucial to investigate the pathological development of KIRC, elucidate its prognostic features, and identify novel therapeutic targets.

Recent studies have shown that KIRC progression is frequently accompanied by significant alterations in necroptosis. Necroptosis, a distinct form of programmed cell death, is mediated by activating key signaling molecules, such as RIPK1, RIPK3 and MLKL) (10, 11). This process results in cell membrane rupture and the release of damage-associated molecular patterns (DAMPs), which stimulate immune responses and link necroptosis to inflammation (12–14). Necroptosis plays a dual role in cancer: in some contexts, it suppresses tumor growth by inducing cell death, while in others, it promotes tumor progression through inflammation and immune modulation. For example, the decrease in RIPK3 expression allows cancer cells to evade necroptosis and correlates with poor prognosis in colorectal cancer (15). Conversely, RIPK1, RIPK3 and MLKL expression levels in hepatocellular carcinoma are linked to improved survival (16). In pancreatic cancer, elevated RIPK1 and RIPK3 levels enhance tumor migration and invasion, while low MLKL expression is associated with worse outcomes (17, 18). In addition to the core biomarkers (*RIPK1, RIPK3*, and *MLKL*), other functionally significant genes, such as *ZBP1, TLR3*, and *PYGL*, are also associated with KIRC progression and play important roles in promoting tumor proliferation during disease development (19–21).

Necroptosis also shapes immune responses within the tumor microenvironment. For instance, RIPK3-mediated PGAM5 activation can boost antitumor immunity via natural killer T cells, independent of necroptosis (22). Additionally, fibroblasts in the tumor microenvironment can drive immune responses through necroptosis-induced NF-κB signaling rather than MLKL-mediated DAMPs release (23). By regulating inflammation, angiogenesis, and metastasis, necroptosis influences both tumor progression and the immune landscape. Understanding these mechanisms is vital for advancing tumor immunotherapy and identifying innovative therapeutic targets.

Compared to other programmed cell death pathways, necroptosis may play a more biologically significant role in KIRC progression. Current research indicates that risk prognostic models based on pyroptosis and ferroptosis have certain limitations and reduced biological relevance, such as the use of relatively small initial gene sets (e.g., n = 60) (24), the lower proportion of differentially expressed genes (DEGs, only 5.29% of total genes) (25), moderate predictive performance (5-year AUC values of 0.68 to 0.76), lack of independent/external validation cohorts (26, 27). In contrast, necroptosis-associated genes can more robustly predict KIRC patient prognosis and show significant correlations with tumor immune microenvironment features, including upregulation of immune checkpoint molecules and regulation of T cell infiltration. These findings highlight their potential as immunotherapy targets and underscore the need to explore alternative biomarkers. However, methodological challenges have greatly hindered the exploration of novel alternative biomarkers, due to the limited scale of available gene sets (<100 genes) and a lack of in-depth mechanistic understanding (3, 26–28). Therefore, expanding necroptosis-related gene sets, validating their predictive performance across diverse populations, and investigating their roles in immune regulation in KIRC are of great scientific and clinical significance.

This study focused on necroptosis-associated genes in the context of KIRC. Using high-throughput computational data, we conducted a complete closed-loop investigation encompassing gene screening → gene expression analysis → model validation → experimental translation. Specifically, we identified necroptosis-related mRNA biomarkers in KIRC samples through transcriptomic analysis, established a risk prognostic model, examined tumor immune microenvironment characteristics and drug responsiveness, and highlighted promising therapeutic candidates along with optimal cell lines for drug-target research. This work not only provides multi-dimensional data to support novel pathological mechanisms of KIRC but also offers valuable insights for developing precise, personalized immunotherapy strategies.

## Materials and methods

### Data source

The initial gene set, comprising 930 necroptosis-associated genes, was obtained from the GeneCards database (https://www.genecards.org/). Based on this gene set, gene expression data—including RNA-seq profiles and associated clinical information—were collected from 542 KIRC tumor tissues and 72 matched normal tissues in The Cancer Genome Atlas (TCGA) database (https://portal.gdc.cancer.gov). Using patient ID numbers, the transcriptome data were matched with clinical information by removing duplicates and mismatched entries. This curation resulted in a final dataset comprising complete gene expression profiles and clinical data for 533 KIRC patients and 72 normal individuals, forming “TCGA cohort”. Clinical information comprised demographic characteristics (sex, age), TNM staging, and survival outcomes (status and overall survival time).

Construction of the risk prognostic model required a “training cohort”, which we derived from gene expression data in TCGA KIRC tumor samples. The genes included in the final risk prognostic model were defined as “parameter genes”. To evaluate the model’s reliability and generalizability, we used an “independent/external validation cohort”. After systematic evaluation of multiple datasets (including ICGC and GEO), we selected the Pan-Cancer Analysis of Whole Genomes (PCAWG) dataset from the UCSC Xena Genomics Platform (https://xenabrowser.net/datapages/) as the independent/external validation cohort. The PCAWG cohort was chosen because (1): it provided complete clinical data including overall survival, unlike other datasets with missing information; (2) By utilizing 68 previously unanalyzed KIRC samples from the PCAWG cohort, we effectively circumvent sample overlap concerns that could arise when using other datasets in conjunction with TCGA samples; (3) it featured uniformly processed gene expression data, ensuring analytical consistency. Furthermore, for the independent external validation cohort, a *post-hoc* power analysis was conducted to assess the adequacy of the sample scale by utilizing the Wilcoxon-Mann-Whitney test implemented in G*Power software (version 3.1.9.7).

To verify the transcriptome data from TCGA tissue samples, we also incorporated candidate cell lines and potential gene targets. The Cancer Cell Line Encyclopedia (CCLE, https://sites.broadinstitute.org/ccle/) and the Human Protein Atlas (HPA, https://www.proteinatlas.org/) provide comprehensive resources. After excluding incomplete samples and cell lines, we obtained 528 TCGA KIRC tumor samples and 23 KIRC-related cell lines, providing robust data for analysis, including uniform manifold approximation and projection (UMAP) matrices from the TCGA cohort, metabolite profiles (225 metabolite levels), gene expression similarity scores, and mRNA/protein expression data. These datasets offer high homogeneity and excellent reproducibility from KIRC-related tumor cell lines, fully meeting the requirements for *in vitro* validation studies.

### Identification of differentially expressed genes

Differential expression analysis was performed on 930 necroptosis-associated genes. Genes with zero expression were excluded, leaving 908 genes for which log2 fold-change (FC) and p-values were calculated. After removing 37 genes lacking unambiguous Ensembl IDs, 574 DEGs were identified using DESeq2, with a significance threshold of FDR (Benjamini-Hochberg adjusted p-value) < 0.001 and |log_2_FC| > 1. Subsequently, Gene Ontology (GO) enrichment analysis was conducted on 551 DEGs with valid Ensembl IDs using the clusterProfiler R package, with the entire annotated protein-coding genome as the background. Gene Set Enrichment Analysis (GSEA) was also performed on 871 necroptosis-associated genes with Ensembl IDs, utilizing genome-wide expression profiles as the background. To ensure statistical robustness, GSEA was restricted to predefined gene sets containing between 10 and 1000 genes. Based on the results for GeneRatio and adjusted p-values (p-adjust), we identified 26 key DEGs that play a significant role in the necroptotic process in KIRC. These key DEGs were prioritized for further investigation into their functional impact and potential as biomarkers or therapeutic targets.

### KEGG-necroptosis pathway DEGs: rendering analysis and PPI analysis

The necroptotic signaling pathway network from the Kyoto Encyclopedia of Genes and Genomes (KEGG) database (http://www.genome.jp/kegg/) were extracted and Pathview R package was used for visualization and analysis. This analysis identified nine core DEGs associated with necroptosis. To explore their intrinsic interactions, we conducted a protein–protein interaction (PPI) network analysis using the STRINGdb R package. The resulting network revealed functional relationships and potential regulatory roles of these core DEGs within the necroptotic signaling pathway.

### Establishment and validation of a risk prognostic model for necroptosis associated genes

To assess the prognostic value of necroptosis-associated genes, we conducted univariate Cox regression analysis on 930 genes associated with necroptosis to evaluate the correlation between genes and survival status in the training cohort. The analysis was performed using the glmnet R package. We applied selection criteria of p-value < 0.05 and a change in hazard ratio (δHR) greater than 0.05% (> 0.05%), identifying 38 candidate genes significantly associated with survival and suitable for prognostic modeling. After analyzing the expression data from the TCGA cohort, the 533 tumor samples were stratified into high-risk and low-risk groups based on risk score analysis. Survival analysis was then conducted using the survminer R package. In addition, a 5-year receiver operating characteristic (ROC) curve was generated using the timeROC R package to evaluate predictive performance. Centroid-based principal component analysis (PCA) was performed with the FactoMineR and factoextra R packages to visualize the distribution of risk groups. To facilitate prognostic modeling, we further employed LASSO regression (using the glmnet R package) and multivariate Cox regression analysis (using the survival R package) to narrow the range of candidate genes, ultimately retaining 6 candidate genes as modeling parameters. In the PCAWG cohort, the normalization of the gene expression matrix, target screening, and other treatments was consistent with that in the TCGA cohort, and all subsequent validation analyses, including risk score analysis, were conducted using only these six determined modeling genes.

### Gene mutation analysis

To conduct a more comprehensive risk assessment for KIRC, we used the maftools R package to analyze mutation profiles of the necroptosis-related gene set, based on gene expression data from high-risk and low-risk groups. The overall mutation landscape of the TCGA KIRC cohort was visualized using data from the iCoMut Beta tool on FireBrowse Platform (http://firebrowse.org/iCoMut/?cohort=KIRC) (29).

### Tumor immune microenvironment analysis

The tumor immune microenvironment analysis was divided into three components: immune infiltration, immune checkpoint expression, and immune phenotyping scores (IPS). To evaluate immune cell infiltration, gene set variation analysis (GSVA) was applied to the expression matrix of the necroptosis-related gene set from 533 tumor samples (high- and low-risk groups). This analysis utilized the “single sample Gene Set Enrichment Analysis (ssGSEA)” tool on the Cloud-Bioinformatics platform (http://www.biocloudservice.com/home.html). Immune checkpoint and immune phenotyping score analyses were performed using the IOBR R package. Data visualization was conducted using the ggplot2 and pheatmap R packages.

### Drug sensitivity analysis

To evaluate the potential therapeutic responsiveness of stratified patient groups, we assessed drug sensitivity in high- and low-risk KIRC cohorts using compounds with established clinical relevance. A total of 37 apoptosis-associated agents were initially retrieved from the Genomics of Drug Sensitivity in Cancer (GDSC) database (https://www.cancerrxgene.org/). From these, nine clinically relevant compounds were selected based on their current or potential therapeutic applications in KIRC treatment. Drug response was predicted using the pRRophetic R package, which estimates the half-maximal inhibitory concentration (IC50) based on gene expression profiles. Comparative analysis of predicted drug sensitivity between risk groups was conducted, and results were visualized using the ggplot2 R package.

### Immunohistochemical analysis

To further clarify the clinical relevance and feasibility of the nine DEGs identified as core markers in KIRC, along with the six parameter genes included in the prognostic risk model, we retrieved immunohistochemical (IHC) staining data for these genes from the HPA database. For sample selection, we prioritized tumor tissues from individuals approximately 60 years of age, ensuring gender consistency across samples. This strategy was informed by the demographic distribution of KIRC cases, in which individuals aged 60 and above accounted for over 54.03% of cases, while those aged 55–65 comprised more than 33.02%.

### Screening for model cell lines

UMAP analysis was first performed on these candidates to achieve dimensionality reduction clustering and visualize differences among samples. Orthogonal-partial least squares discriminant analysis [(O)PLS-DA] was performed on 23 KIRC-related cell lines, utilizing three inputs: cluster consistency results, risk analysis outcomes, and a gene expression similarity matrix. The resulting VIP values were utilized for downstream selection of candidate cell lines, with a threshold of average VIP score > 1 (calculated by integrating both cluster- and risk-VIP scores). The candidate cell lines underwent differential analyses of gene expression and metabolite profiles. To further prioritize targets, paired mRNA and protein expression profiles from HPA database were utilized to identify top candidate cell lines and necroptosis-related potential gene targets for experimental validation.

## Results

### Screening of DEGs and analysis of their interaction networks

**Figure 1:**
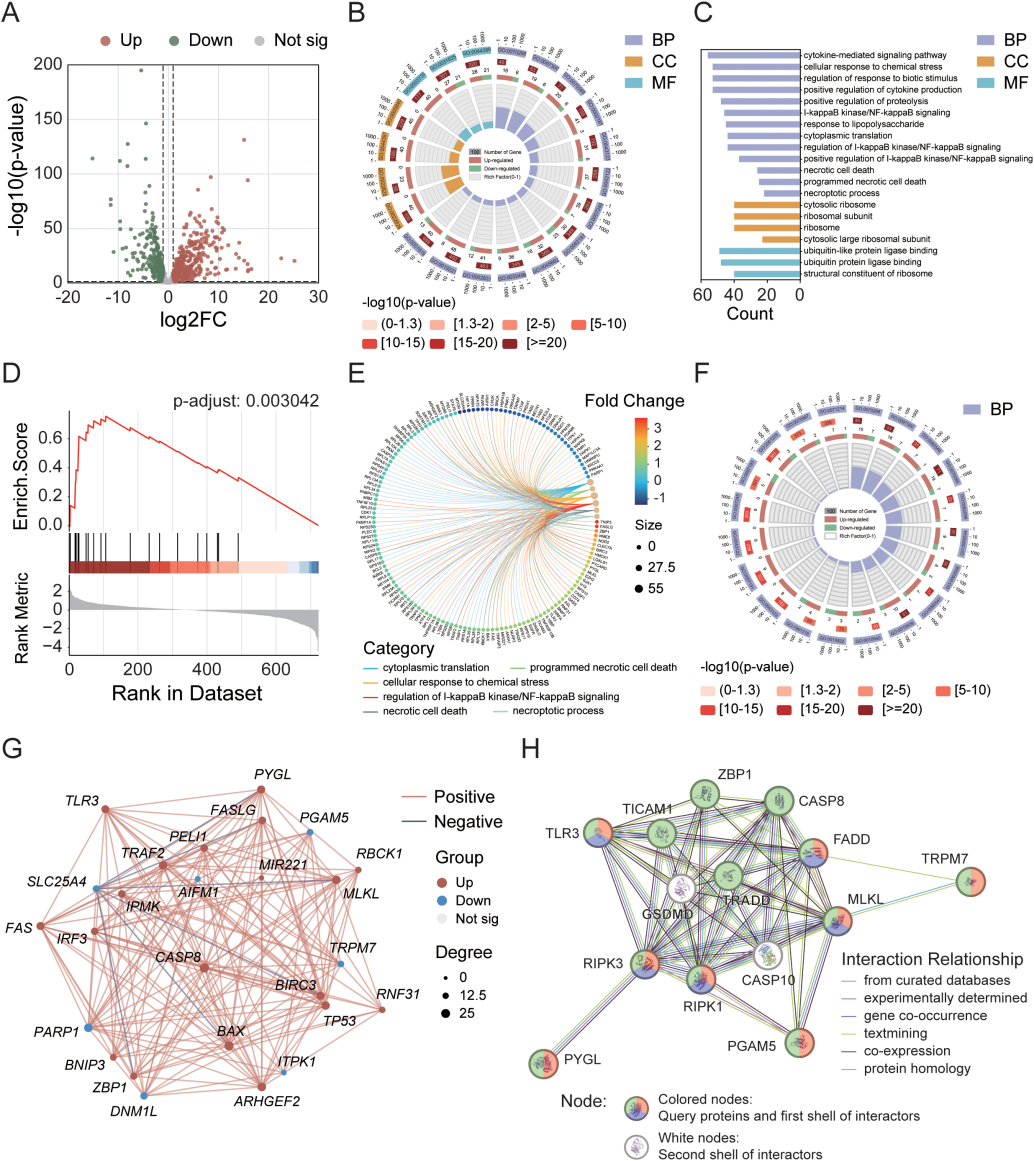
Identification of differentially expressed genes (DEGs) in the TCGA cohort and analysis of their interaction networks. **(A)** Volcano plot showing DEGs within the necroptosis gene set. **(B)** Circular diagram illustrating results of GO enrichment analysis. **(C)** GO enrichment analysis of 574 necroptosis-related DEGs. **(D)** GSEA enrichment analysis highlighting key pathways. **(E)** Correlation analysis between DEGs and metabolic pathways. **(F)** GO enrichment analysis of 26 key DEGs. **(G)** Interaction network of the 26 key DEGs. **(H)** PPI network analysis of 9 core DEGs. Node colors represent different annotations: green—KEGG necroptosis pathway (hsa04217); red—biological processes associated with necroptosis (GO:0070266); purple—necroptotic signaling pathway (GO:0097527).

In the GeneCards database, we obtained 930 genes associated with the process of necroptosis. We identified 343 upregulated and 231 downregulated DEGs on the basis of the criteria of p-value < 0.001 and |log_2_FC| > 1 (**Figure 1A**). We subsequently conducted enrichment analysis on these DEGs. The GO enrichment results revealed that the enrichment rates for biological processes (BP) related to the necroptotic process, programmed necrotic cell death, and necrotic cell death, as well as for cellular components (CC), such as the cytosolic ribosome and the cytosolic large ribosomal subunit, exceeded 30%. In terms of the absolute number of genes enriched, BPs such as the cytokine-mediated signaling pathway, the cellular response to chemical stress, the regulation of the response to biotic stimulus, and the positive regulation of cytokine production were enriched in more than 50 genes, but the enrichment rates were less than 20% (**Figures 1B, C**). GSEA further revealed 21 gene functional groups that met specific criteria (NES > 1, p-adjust < 0.05, enrichment score > 0), accounting for approximately 0.25% of the GSEA classifiable gene functional groups, which could be categorized into related disease categories such as kidney diseases, neurological disorders, and muscle and movement disorders (**Figure 1D**). To identify key DEGs involved in the process of necroptosis, we conducted a secondary enrichment analysis (to distinguish this analysis from the GO analysis of 574 DEGs shown in **Figure 1B**). This analysis ultimately identified 26 key DEGs (**Figures 1E, F**). Through analysis of the interaction network (**Figure 1G**), we found that compared with the normal group, the tumor group had 19 genes whose expression was significantly upregulated and 7 genes whose expression was significantly downregulated, both with p-value < 0.05. Furthermore, through KEGG-necroptosis signaling pathway rendering analysis (**Supplementary Figure 1**), we identified 9 core DEGs, including *RIPK1*, *RIPK3*, and *MLKL*. Although *RIPK1* and *RIPK3* were not among the 26 key DEGs, they were still classified as core genes due to their indispensable roles in necroptosis. Other core DEGs included *CASP8*, *ZBP1*, *TLR3*, *PYGL*, *TRPM7*, and *PGAM5*. Finally, we conducted a PPI analysis on these 9 core DEGs to elucidate their interactions (**Figure 1H**).

### Tumor sample typing and survival analysis based on DEGs

**Figure 2:**
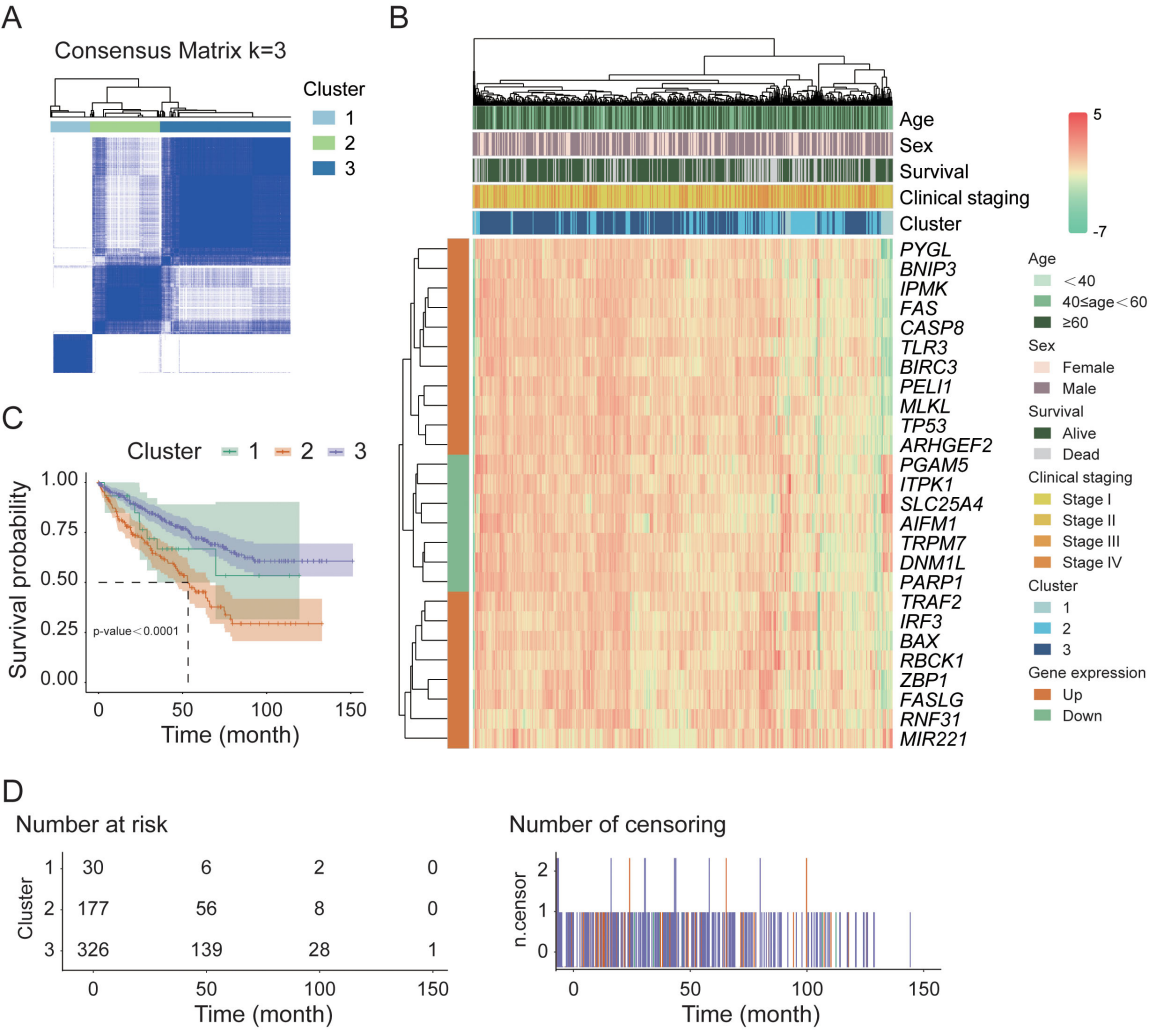
Sample classification and survival analysis in the TCGA cohort. **(A)** Consensus clustering analysis of normal and tumor samples based on necroptosis-related DEGs. **(B)** Hierarchical clustering heatmap of 26 key DEGs in tumor samples. **(C)** Kaplan–Meier survival analysis of tumor samples stratified by consensus clustering results. **(D)** Distribution of tumor sample counts across different survival time points.

To explore the relationships between the 26 DEGs associated with necroptosis and the subtypes of KIRC, we performed a consistency clustering analysis on 72 normal and 542 tumor samples (**Figure 2A**). By incrementally increasing the value of the clustering variable “k”, we find that when k=3, the similarity within groups is the highest, and the similarity between groups is the lowest. These findings indicate that these samples could be divided into three distinct clusters (C1-C3) on the basis of the 26 DEGs. We subsequently conducted a heatmap analysis of tumor samples to visualize the gene expression profiles and clinical characteristics of these samples, including age, sex, survival status, and clinical staging information (**Figure 2B**). The results revealed that, under the influence of various clinical factors, there were no significant differences in the clinical characteristics among the 533 samples. However, in terms of gene function regulation, the upregulated and downregulated genes presented distinct clustering characteristics. Further survival analysis of these three clusters (**Figures 2C, D**) revealed a highly significant difference in survival rates between the clusters (p-value < 0.0001). The survival rates were 20.0%, 31.6%, and 42.6% for clusters C1, C2, and C3, respectively, during the 50-month follow-up period.

### Establishment of a risk prognostic model in the TCGA cohort

**Figure 3:**
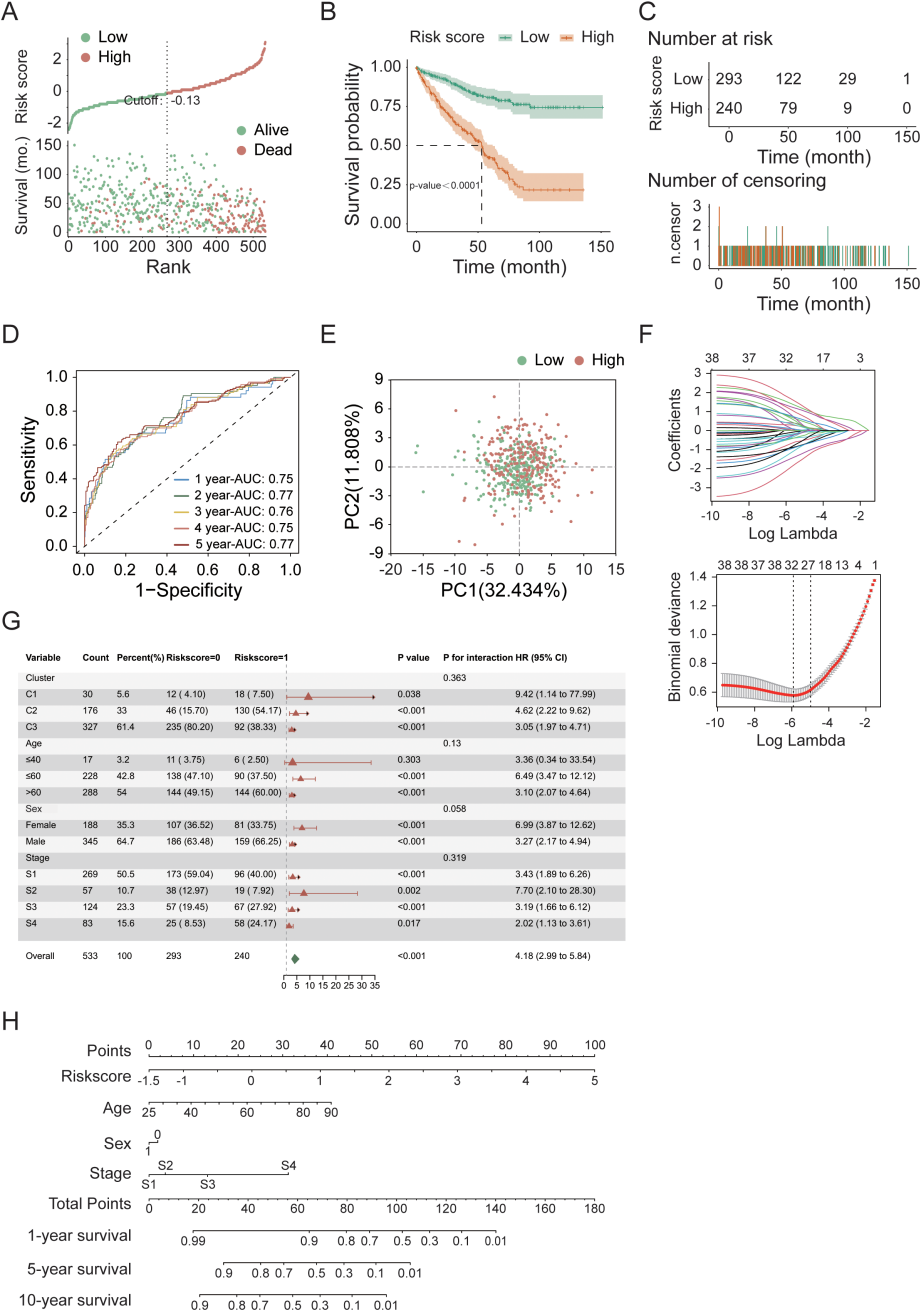
Prognostic modeling and convergence analysis of feature genes in the TCGA cohort. **(A)** Risk score distribution of tumor samples. **(B)** Kaplan–Meier survival analysis comparing high- and low-risk groups. **(C)** Temporal distribution of tumor samples in high- and low-risk groups over survival time. **(D)** Time-dependent ROC (timeROC) curve analysis for 5-year survival prediction. **(E)** Principal component analysis (PCA) of high- and low-risk groups. **(F)** Convergence analysis of candidate prognostic genes using LASSO regression and multivariate Cox regression. **(G)** Subgroup survival analysis of high- and low-risk groups (additional results shown in **Supplementary Figure 2**). **(H)** Nomogram-based risk score analysis of tumor samples.

To explore the prognostic value of necroptosis-associated genes, we first standardized the tumor sample data from 533 patients in the TCGA cohort whose complete gene expression and clinical information were available. We subsequently applied LASSO-Cox regression analysis to identify 38 candidate genes associated with survival that are suitable for prognostic modeling, using selection criteria that included a p-value less than 0.05 and a change in the hazard ratio (HR) greater than 0.05% (δHR > 0.05%). After performing risk analysis (**Figure 3A**), we identified 240 high-risk samples and 293 low-risk samples. Compared with the other groups, the low-risk group had a longer survival time and a lower mortality rate. On the basis of this risk stratification, we subsequently conducted intergroup survival analysis, time-dependent receiver operating characteristic (timeROC) curve analysis, and centroid PCA (**Figures 3B–E**). The results revealed a highly significant difference between the high-risk and low-risk groups (p-value < 0.0001). Additionally, the area under the timeROC curve from 1–5 years indicated no significant difference in the sensitivity of the test samples over this period. The centroid PCA results revealed that the sample distribution showed little difference. To optimize the model and account for the correlation between gene functional expression, we performed a secondary convergence analysis using LASSO regression and multivariate Cox regression on the candidate genes for modeling (**Figure 3F**, **Table 1**, **Supplementary Table 1**, **Supplementary Table 2**), ultimately retaining 6 candidate genes (*IL4*, *CDC7*, *IGF2BP3*, *CASP9*, *TYRO3*, and *CPT2*) as modeling parameters (multivariate Cox regression screening threshold: p-value < 0.05). The expression of the multivariate Cox regression model is as follows: $f(t|x)=f_{0}(t)\times \exp(coef_{1}\cdot gene_{1}+coef_{2}\cdot gene_{2}+\cdots +coef_{n}\cdot gene_{n})$ , where f(t|x) is the risk function at time; $f_{0}(t)$ is the baseline risk function; $coef_{n}$ is the coefficient of each predictive variable factor; and $gene_{2}$ is the necroptosis-associated gene affecting survival. In this model, the risk score can be expressed as $f\left(t|x\right)=f_{0}\left(t\right)\times \text{exp}\left(0.8940\cdot IL4+1.5176\cdot CDC7+0.4019\cdot IGF2BP3+1.8612\cdot CASP9-0.8737\cdot TYRO3-1.0633\cdot CPT2\right)$ Finally, we conducted subgroup analysis on other factors in the model (**Figure 3G**, **Supplementary Figure 2**). Although there were no significant differences in clinical factors between the high- and low-risk groups, after each factor was subgrouped, the differences between the high- and low-risk groups reached significant (p-value < 0.05) or highly significant levels (p-value < 0.01). Considering the risk score, age, sex, and clinical stage of the patients, we found that the total risk score (**Figure 3H**) could serve as an important indicator for predicting survival rates. When the score is less than 80, the expected one-year survival rate for patients exceeds 80%, and the five-year survival rate is approximately 30%.

**Table 1:** Results of multivariate Cox analysis (p-value < 0.05).

Gene ID	Coef	HR	HR.95 L	HR.95H	p-value
*IL4*	0.8940	2.4448	1.3065	4.5751	0.0052
*CDC7*	1.5176	4.5612	1.4920	13.9442	0.0078
*IGF2BP3*	0.4019	1.4946	1.0951	2.0400	0.0113
*CASP9*	1.8612	6.4312	1.4640	28.2512	0.0137
*TYRO3*	-0.8737	0.4173	0.1846	0.9439	0.0359
*CPT2*	-1.0633	0.3453	0.1268	0.9407	0.0376

### External validation of the risk prognostic model in the PCAWG cohort

**Figure 4:**
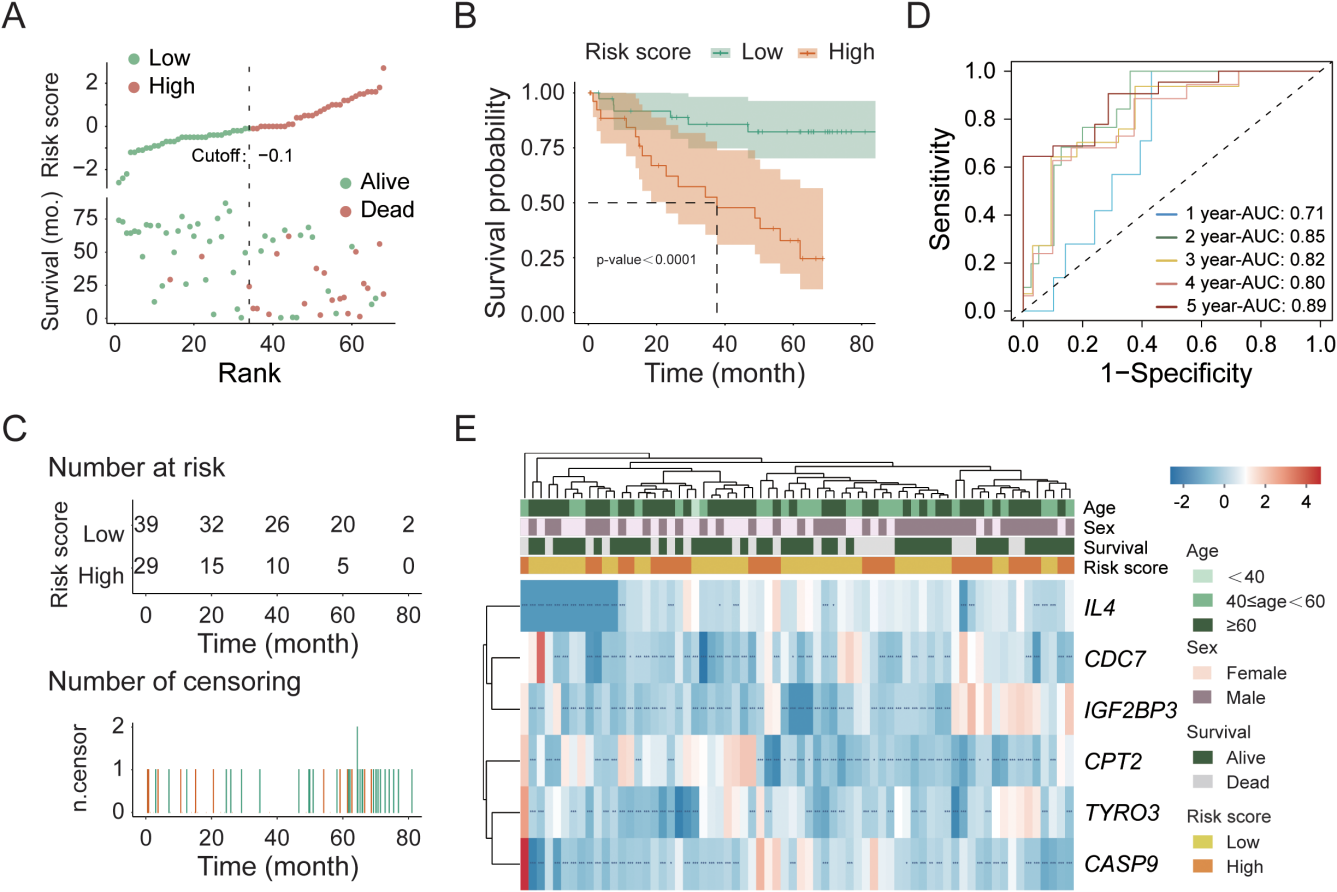
Validation of the prognostic model in the PCAWG cohort. **(A)** Risk score distribution of tumor samples. **(B)** Kaplan–Meier survival analysis of high- and low-risk groups. **(C)** Temporal distribution of high- and low-risk groups over survival time. **(D)** Time-dependent ROC (timeROC) curve analysis for 5-year survival. **(E)** Hierarchical clustering heatmap of the six model genes in tumor samples.

For external validation, we utilized 68 KIRC patient samples from the PCAWG cohort in the UCSC database. Before conducting the sample analysis, we performed the same data normalization and filtering as with the TCGA cohort, and the gene matrix included expression data for only the 6 genes used for modeling. Using risk score analysis (**Figure 4A**), we categorized the samples into a high-risk group (29 cases) and a low-risk group (39 cases). The results indicated that patients in the low-risk group presented longer survival times and lower mortality rates than did those in the high-risk group. Further survival analysis (**Figures 4B, C**) revealed a highly significant difference (p-value < 0.0001) between the high- and low-risk groups. Additionally, the area under the curve (AUC) of the 5-year timeROC analysis exceeded 0.89, which demonstrates the good predictive performance of our model (**Figure 4D**). Finally, we performed a heatmap clustering analysis of gene expression and clinical information (**Figure 4E**), revealing that the 6 modeling genes were primarily grouped into two clusters: one consisting of *IL4*, *CDC7*, and *IGF2BP3* and the other consisting of *CASP9*, *TYRO3*, and *CPT2*. The *post-hoc* statistical power analysis (**Table 2**) confirmed that the PCAWG cohort had strong sensitivity for detecting clinically significant differences, with a power (1 − β error probability) of 0.9999, exceeding the commonly accepted threshold of 0.9.

**Table 2:** *Post-hoc* statistical power analysis based on ROC-AUC (PCAWG cohort).

Item	No.	Parameters	Value	Explanation
Input	1	Tail (s)	One	
	2	Parent distribution	Normal	
	3	α err prob	0.05	
	4	Effect size d*	/	Automated export
		◼ Mean group 1 (Low-risk group)	-0.659	Risk score
		◼ Mean group 2 (High-risk group)	0.887	
		◼ SD σ within each other(*SD_pooled_*)	0.636	Formula-derived
	5	Sample size group 1 (Low-risk group)	20	Number of 50% samples
	6	Sample size group 2 (High-risk group)	15	
Output	7	Noncentrality parameter δ	6.956	
	8	Critical t	1.695	
	9	Df	31.423	
	10	Power (1-β error probability)	0.9999	

*Effect size d: was automatically computed by the analytical software, incorporating the mean group 1, mean group 2, and the pooled standard deviation (*SD_pooled_*). The *SD_pooled_* was from following Cohen’s d formula:
$$SD_{pooled}=\sqrt{\frac{\left(n_{Low}-1\right)\times SD_{Low}^{2}+\left(n_{High}-1\right)\times SD_{High}^{2}}{n_{Low}+n_{High}-2}}$$

n: sample size. *SD*, standard deviation.

### Mutation analysis of necroptosis gene sets in the TCGA cohort

**Figure 5:**
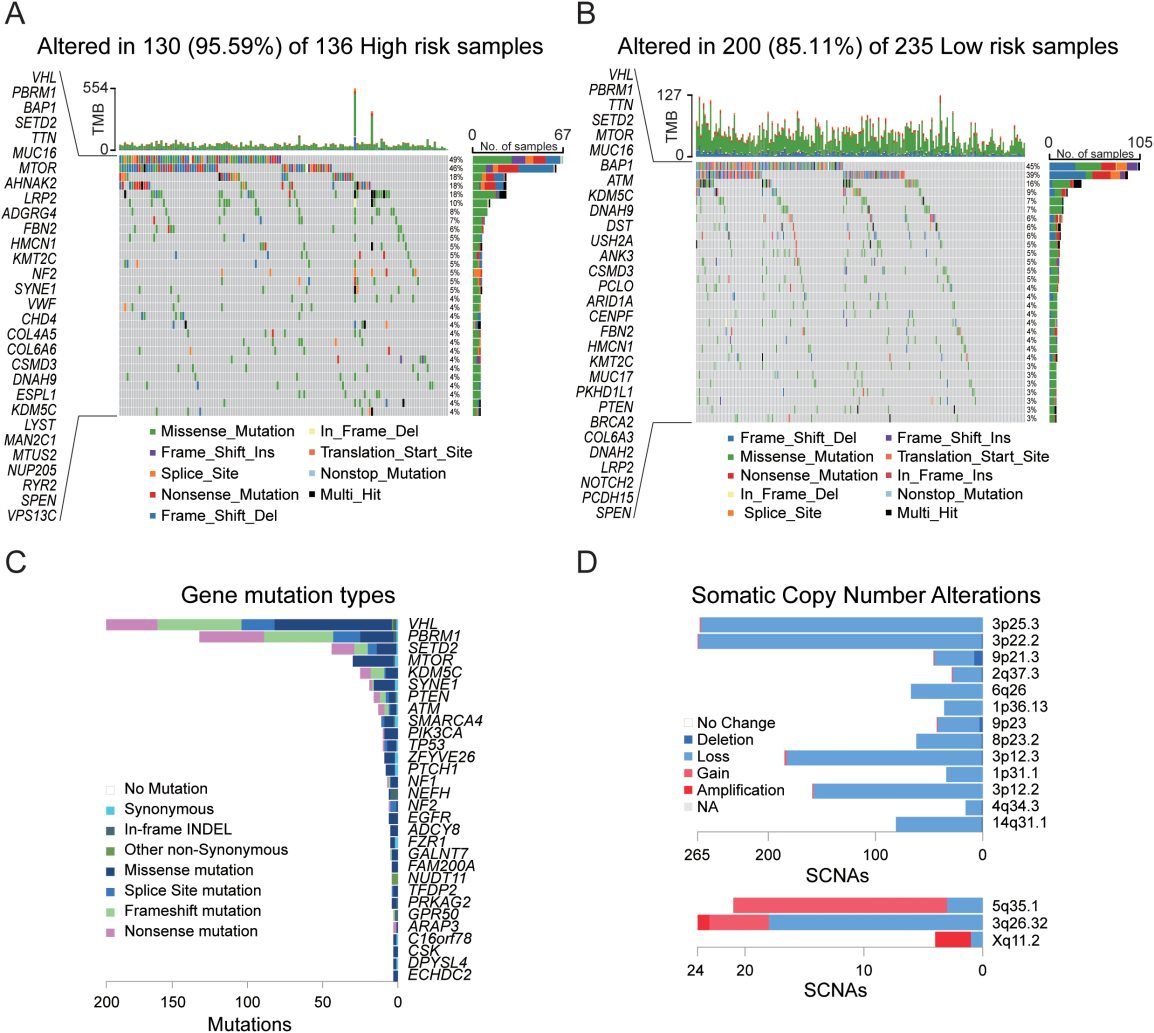
Gene mutation analysis in the TCGA cohort. **(A)** Mutation landscape in tumor samples from the high-risk group. **(B)** Mutation landscape in tumor samples from the low-risk group. **(C)** Summary of gene mutation types and chromosomal localization across 533 tumor samples. **(D)** Chromosomal localization and somatic copy number alterations (SCNAs) across 533 tumor samples.

To conduct a more comprehensive risk assessment for KIRC, we utilized the TCGA cohort to perform mutation analysis on the necroptosis gene set. The analysis results (**Figures 5A, B**) revealed that among the high-risk and low-risk samples, 136 and 235 samples, respectively, could be matched with the mutation database, with matching rates of 56.67% and 80.20%. In the matched samples, the mutation rates for the high-risk group and the low-risk group were 95.59% and 85.11%, respectively. The primary mutated genes in both groups were *VHL* and *PBRM1*, with mutation rates reaching 40%. The *VHL* gene primarily affects tumor development by regulating the hypoxia response, whereas the *PBRM1* gene contributes to tumor development through chromatin remodeling and immune regulation. The combined loss of both genes synergistically promotes the occurrence of renal cancer. The secondary mutated genes include *SETD2* and *TTN*, with a mutation rate of 10%. Additionally, *BAP1* and *MUC16* are secondary mutated genes in high-risk samples. The comprehensive analysis of sample mutations (**Figures 5C, D**, **Supplementary Figure 3**) revealed that chromosomal mutation copy losses were primarily concentrated at the 3p25.3, 3p22.2, and 3p12.3 loci, whereas copy gains were primarily concentrated at the 5q35.1 locus. These loci are related to the occurrence and development of tumors, particularly renal cell carcinoma. The genetic changes at these loci are closely associated with the biological behavior and prognosis of the tumor.

### Tumor immune microenvironment analysis

**Figure 6:**
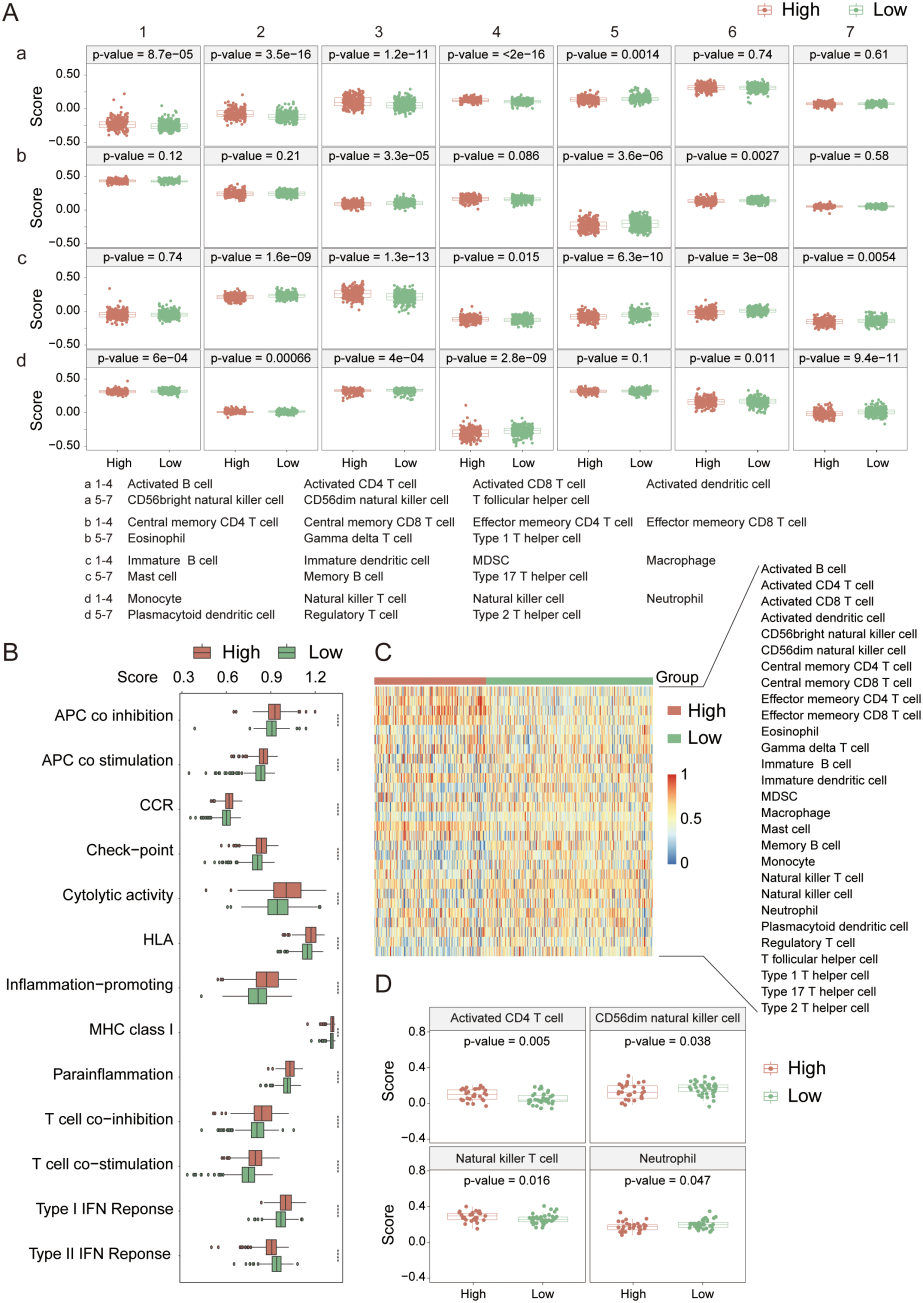
Immune cell infiltration analysis in the TCGA cohort. **(A)** Comparison of infiltration levels for 28 immune cell types between high- and low-risk groups. **(B)** Comparison of 13 immune-related signaling pathways between high- and low-risk groups. **(C)** Heatmap showing the infiltration levels of 28 immune cell types in high- and low-risk groups. **(D)** Immune cell infiltration analysis in the PCAWG cohort. *indicates p-value < 0.05; **indicates p-value < 0.01; ***indicates p-value < 0.001; ****indicates p-value < 0.0001.

To delve deeper into the interactions between KIRC and the immune system, we initially conducted an immune infiltration analysis on the high-risk and low-risk groups within the TCGA cohort. The analysis revealed significant differences in 21 out of 28 immune cell types (**Figure 6A**) between the two groups (p-value < 0.05), with 15 showing extremely significant differences (p-value < 0.001). Specifically, the high-risk group typically exhibited increased levels of immune cell infiltration (**Figure 6C**), particularly in activated B cells, activated CD4^+^ T cells, activated CD8^+^ T cells, activated dendritic cells (DCs), and myeloid-derived suppressor cells (MDSCs). Further analysis of 13 immune signaling pathways (**Figure 6B**) revealed all pathways presented extremely significant differences between the high- and low-risk groups (p-value < 0.001), with the high-risk group generally having a greater infiltration capacity in these pathways. These results suggest that in terms of the immune response, the high-risk group generally has a stronger immune response capability than the low-risk group does, which has significant implications for the prognosis and therapeutic response of KIRC patients.

In the PCAWG cohort (**Figure 6D**), significant differences were observed in the infiltration of activated CD4^+^ T cells, CD56dim natural killer (NK) cells, natural killer T (NKT) cells, and neutrophils between the high- and low-risk groups (p-value < 0.05). Compared with the TCGA cohort, the high-risk cohort presented a greater infiltration capacity for activated CD4^+^ T cells and NKT cells, and the differences in activated CD4^+^ T cells and NKT cells between the groups were even more pronounced (p-value < 0.02). Therefore, we believe that the high-risk group in the PCAWG cohort possesses stronger immunogenicity and higher activity in terms of immune cell infiltration. The lack of prominent characteristics in the data may be attributed to the small sample size.

**Figure 7:**
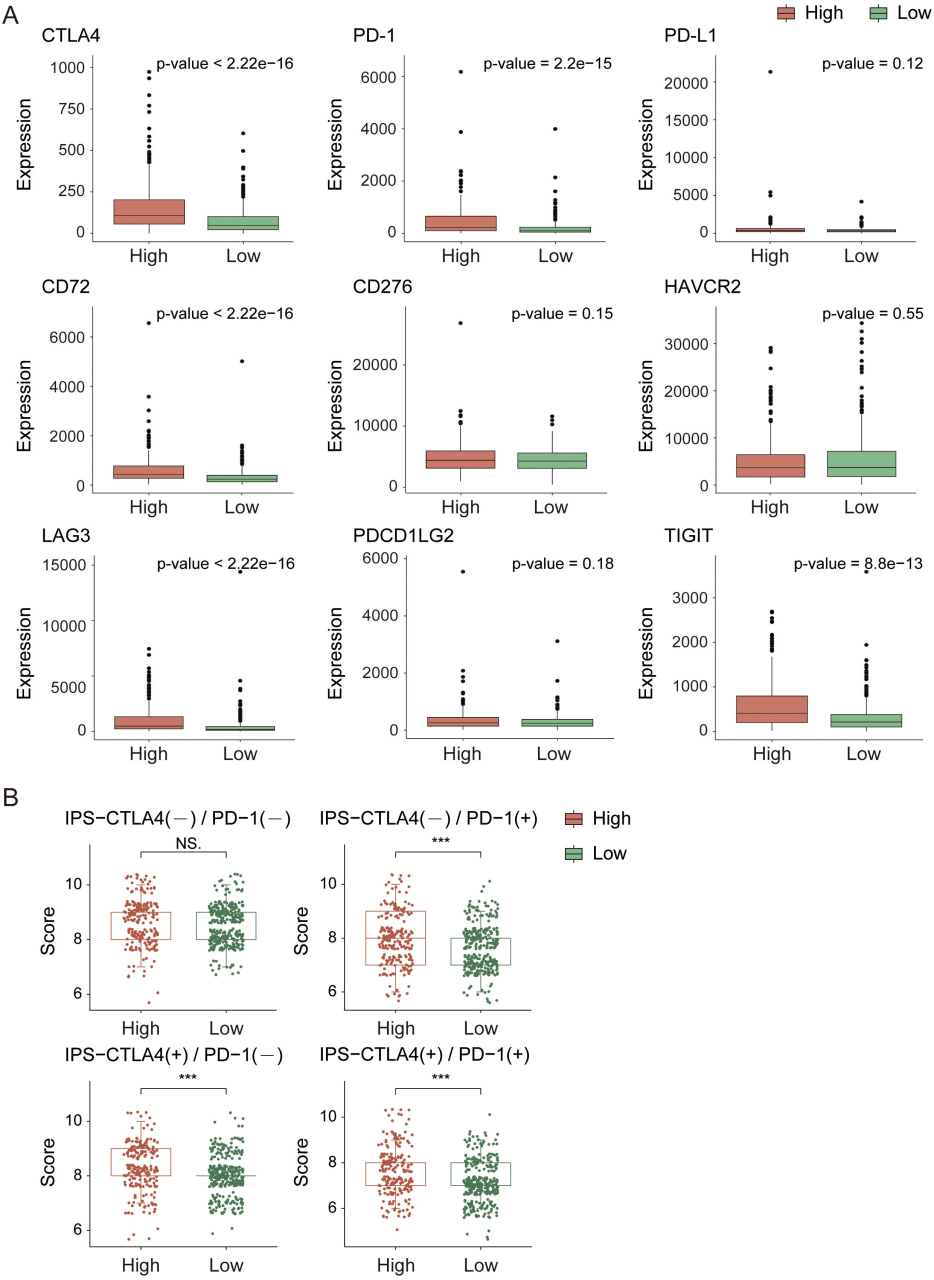
Immune checkpoint and immune score analysis in the TCGA cohort. (**A**) Expression analysis of immune checkpoint genes. **(B)** Immune score comparison between high- and low-risk groups. *indicates p-value < 0.05; **indicates p-value < 0.01; ***indicates p-value < 0.001.

Next, we analyzed the immune checkpoint and immune phenotype scores (IPS) of the samples. Among the nine immune checkpoints closely associated with KIRC (**Figure 7A**), the expression levels of immune checkpoints were generally lower in the low-risk group. In comparisons between the groups, the expression levels of CTLA4, PD-1, CD72, LAG3, and TIGIT were extremely significantly different (p-value < 0.0001). According to the immune phenotype score analysis (**Figure 7B**), there was no significant difference in scores between the high-risk and low-risk groups when there was no response to either CTLA4 or PD-1 antibodies (p-value > 0.05). However, when there was a response to either or both antibodies, there was an extremely significant difference in scores between the groups (p-value < 0.001), with the high-risk group scoring higher than the low-risk group. This result further confirms that the high-risk group has a greater degree of immune cell infiltration and stronger immunogenicity and suggests that the tumor microenvironment in the high-risk group may be more active and potentially more responsive to immunotherapy.

### Analysis of drug response sensitivity based on gene expression matrices

**Figure 8:**
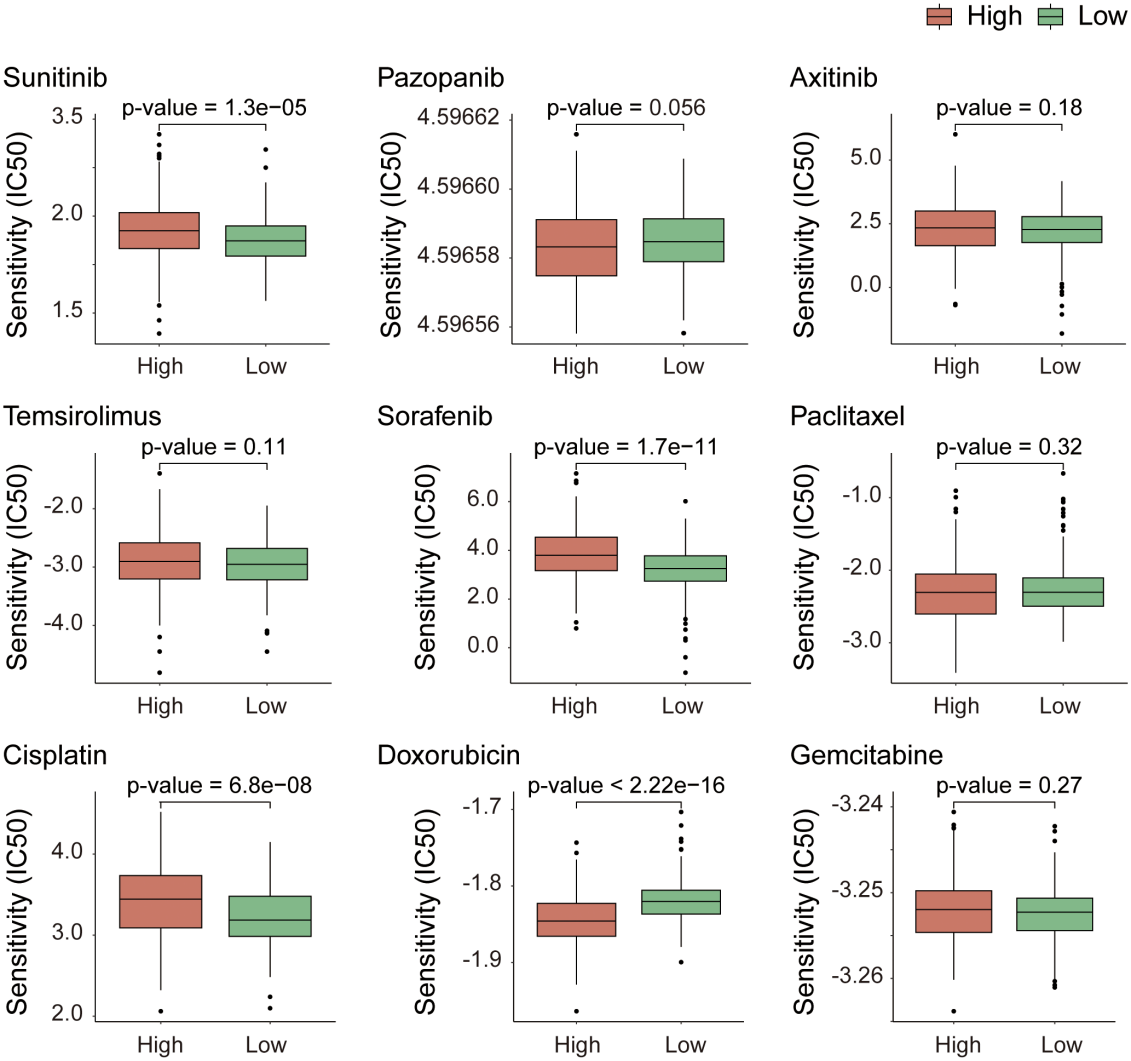
Drug response sensitivity analysis based on gene expression profiles. Prediction of chemotherapeutic and targeted drug sensitivity according to the expression matrix of risk model genes.

To predict how different patient populations, respond to drugs and to identify subgroups that respond well to specific drugs, thereby obtaining more targeted treatment plans, we conducted a drug sensitivity analysis on both high- and low-risk groups. For the analysis, we selected drugs commonly used to treat advanced KIRC, including sunitinib, pazopanib, axitinib, and temsirolimus; sorafenib and paclitaxel for KIRC treatment; and cisplatin, doxorubicin, and gemcitabine, which can treat a variety of cancers. The analysis results (**Figure 8**) revealed extremely significant differences in the response to sunitinib, sorafenib, cisplatin, and doxorubicin between the high- and low-risk groups (p-value < 0.001). With the exception of doxorubicin, the other three drugs exhibited stronger sensitivity in the low-risk group and stronger resistance in the high-risk group. Compared with drugs, doxorubicin has the highest sensitivity, followed by sunitinib. This finding suggests that in the process of treating KIRC, in addition to sunitinib, which is currently the most widely used agent, doxorubicin also holds potential as a therapeutic agent for KIRC.

### Analysis of immunohistochemical staining based on gene expression

**Figure 9:**
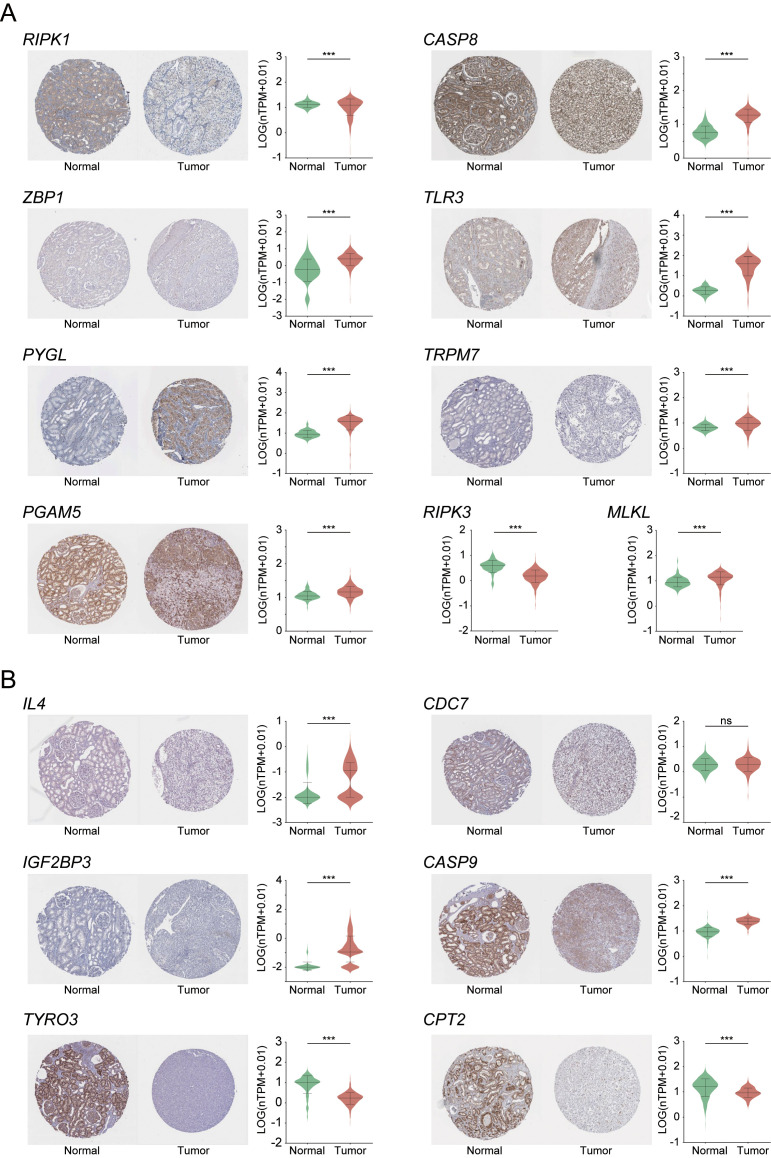
Immunohistochemical analysis of necroptosis-related core DEGs **(A)** and six risk prognostic genes **(B)** in KIRC. Expression levels are shown as normalized transcripts per million (nTPM). *RIPK3* and *MLKL* are excluded from panel A due to unavailable IHC data. ***indicates p-value < 0.001.

Based on the above findings, we further evaluated the clinical relevance of the nine core genes and the six genes included in the prognostic risk model by analyzing their IHC staining patterns in KIRC (**Figure 9**). The analysis revealed that most core genes exhibited consistent expression trends between normal and tumor tissues. Notably, *RIPK1* and *RIPK3* were highly expressed in normal tissues, suggesting their potential roles as tumor suppressors in KIRC progression. In contrast, the remaining seven core genes showed elevated expression in tumor tissues, indicating possible oncogenic functions. Among the prognostic risk model genes, *TYRO3* and *CPT2* demonstrated strong tumor-suppressive potential, aligning with mRNA-level analyses in which both genes showed negative correlation coefficients within the risk model—further supporting their protective roles in KIRC.

### Screening analysis of model cell lines

**Figure 10:**
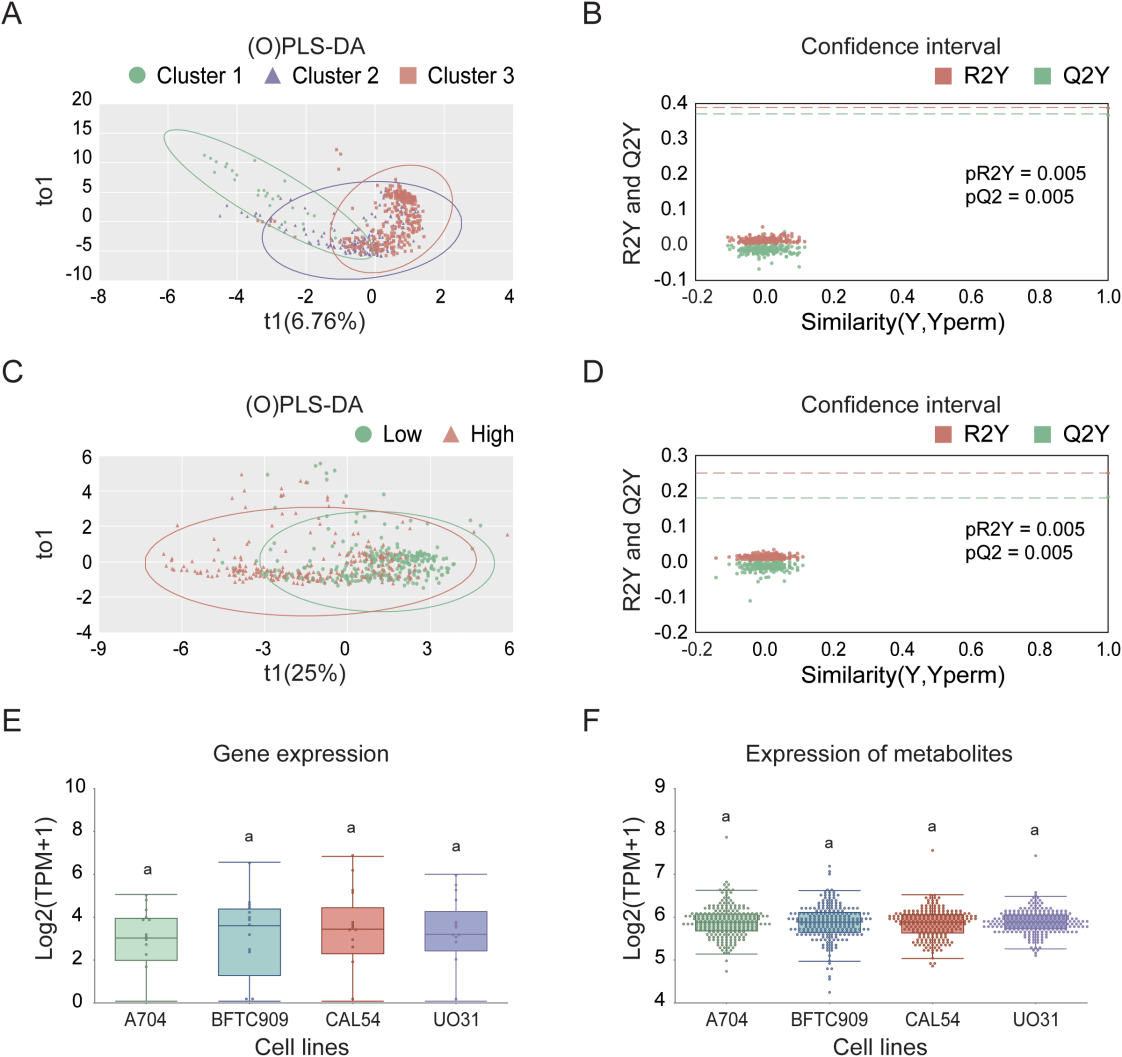
Selection and characterization of KIRC model cell lines in the TCGA cohort. (**A**) (O)PLS-DA based on consensus clustering results. **(B)** Validation of (O)PLS-DA model using permutation testing. **(C)** (O)PLS-DA based on high- and low-risk group classification. **(D)** Confidence test for (O)PLS-DA in high- and low-risk groups. **(E)** Differential expression analysis of model cell lines. **(F)** Analysis of differentially abundant metabolites in selected model cell lines.

In the process of searching for cell lines suitable for modeling KIRC, we first employed UMAP analysis for dimensionality reduction, clustering, and visualization of the TCGA cohort tumor samples. This analysis revealed significant differences among the samples, as shown in **Supplementary Figure 4**. The results indicated that sex did not affect the spatial distribution of the three subtypes: C1, C2, and C3. Specifically, the C2 and C3 subtypes presented closely related spatial positions, whereas the C1 subtype presented a unique distribution characteristic. We subsequently screened 23 candidate cell lines and, through (O)PLS-DA), found that the similarity score matrix of gene expression patterns displayed distinct clustering differences among subgroups within groups, with smaller differences within subgroups, as depicted in **Figures 10A, C**. In the confidence test analyses, as shown in **Figures 10B, D**, Q2Y and R2Y represent the prediction rate and explanation rate of the model, respectively. Although both values were less than 0.4, the predicted values were consistently lower than the original values, and both pQ2Y and pR2Y were less than 0.05, indicating a certain degree of reliability and stability for the model. Finally, based on the VIP average values from the (O)PLS-DA (where the absolute VIP values were greater than 1, as detailed in **Table 3** and **Supplementary Table 3**), we selected four cell lines, A704, BFTC909, CLA54, and UO31, as model cell lines. The differential analysis of gene expression and metabolite levels revealed no significant differences among these four cell lines (**Figures 10E, F**), allowing subsequent research to choose different cell lines for validation studies on the basis of specific research characteristics.

**Table 3:** VIP values of the four model cell lines analyzed by (O)PLS-DA.

Cell lines	Cluster-VIP	Risk-VIP	Average-VIP
A704	3.3033	1.1168	2.2101
BFTC909	1.6551	1.2100	1.4326
CAL54	1.3201	0.9087	1.1144
UO31	1.6238	1.0257	1.3248

**Figure 11:**
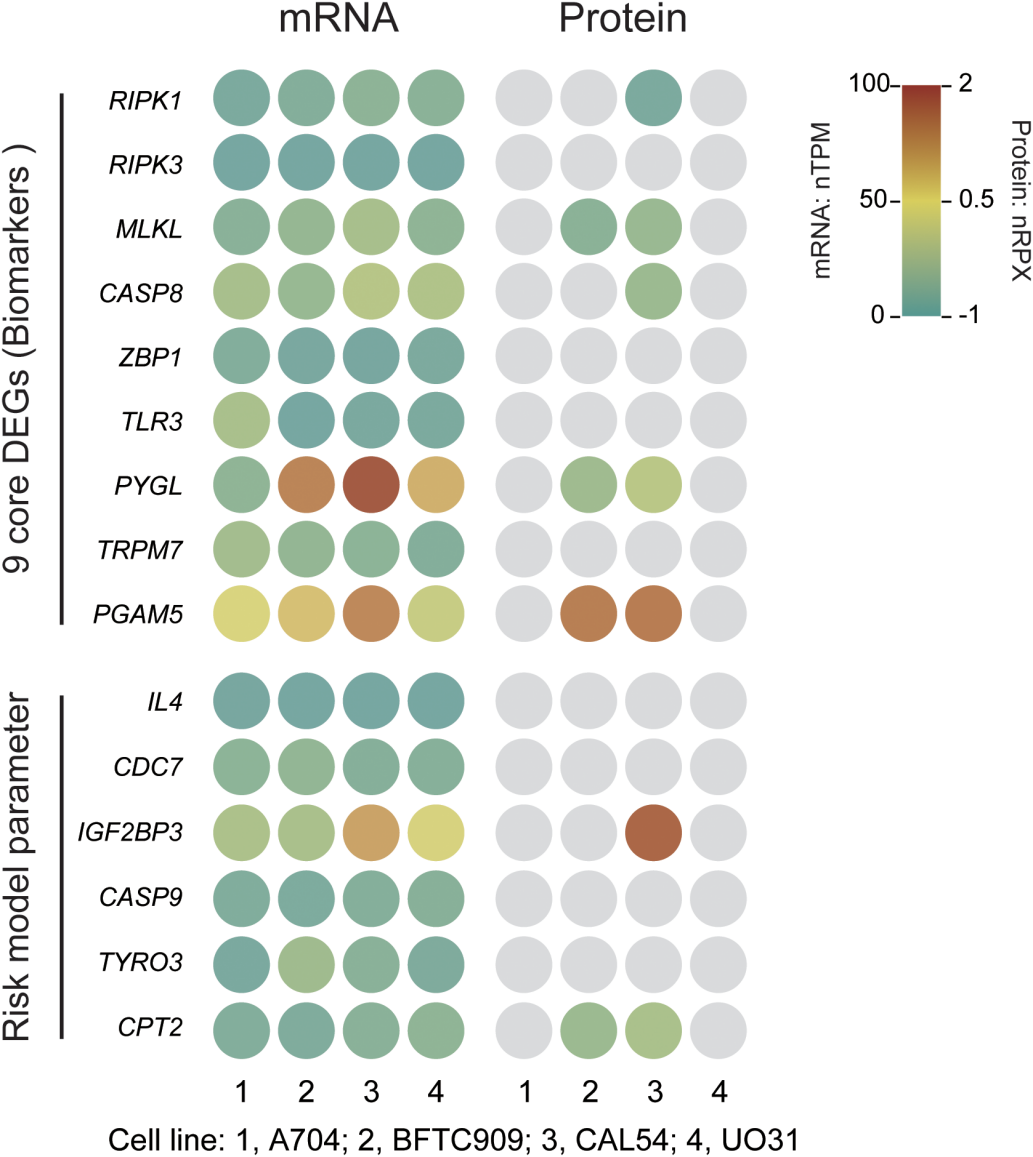
Expression of necroptosis-related core genes and risk model parameter genes in KIRC cell lines. mRNA expression is presented as normalized transcripts per million (nTPM), and protein expression as normalized relative protein expression (nRPX, log2 scale). Grey circles indicate undetected or missing protein data (Pan-Cancer Atlas MS data).

Finally, we used BFTC909 and CAL54 as the optimal cell-line models for dissecting phenotypic heterogeneity in mechanistic and drug-development studies, and we highlight three novel targets (*CASP8*, *PGAM5*, and *CPT2*) for further investigation (**Figure 11**). Three independent lines of evidence support this conclusion (1). An integrated analysis of core/risk prognostic gene expression (mRNA and protein) with cluster-VIP and risk-VIP profiles ranked the lines BFTC909 > CAL54 > A704 > UO31 (**Table 3**; **Supplementary Table 3**). (2) *CASP8* and *PGAM5* displayed stable RNA-protein co-expression in BFTC909 and CAL54, confirming that these lines faithfully recapitulate key pathway activity. (3) *CPT2* showed marked transcript-protein discordance (high mRNA, low protein), offering a unique system for studying post-transcriptional regulation. Together, our analytical framework—which combines cell-line-specific profiling, clinical-risk stratification (cluster- and risk-VIP), and multi-omics validation (RNA [nTPM]-protein [nRPX] concordance analysis) - not only enabled robust model selection but also systematically prioritized targets with both biological significance and clinical translatability.

## Discussion

KIRC is one of the most prevalent subtypes of RCC, characterized by heterogenous cancers of renal tubular epithelial origin (2). It is often associated with mutations in the *VHL* gene, leading to overexpression of HIF1 and HIF2 or the silencing of other tumor suppressor genes (5, 7, 8). Despite advances in treatment, KIRC remains associated with high mortality and tumor metastasis (2, 30, 31). Necroptosis, a regulated form of cell death distinct from apoptosis and necrosis, is mediated by *RIPK1*, *RIPK3*, and *MLKL* (10, 32, 33). It plays a critical role in tumor progression and immune regulation by inducing cell membrane rupture and releasing DAMPs (18). Meanwhile, necroptosis activates immune responses via the NF-κB pathway, further linking it to tumor immunity (23, 34). Given the critical role of necroptosis in tumor progression and immune regulation, recent research has increasingly focused on its potential as a prognostic biomarker and therapeutic target in KIRC.

Recent evidence indicates that necroptosis-related biomarkers may surpass other programmed cell death pathways in predicting KIRC prognosis and informing immunotherapy approaches. Compared with pyroptosis- or ferroptosis-based models, necroptosis signatures exhibit greater prognostic reliability in KIRC and show stronger associations with immune microenvironment characteristics, such as immune checkpoint upregulation and regulatory T cell infiltration (24, 25). However, existing studies remain constrained by the use of limited gene panels and a lack of comprehensive mechanistic investigation (3, 26–28). Future research should prioritize expanding necroptosis-related gene sets, validating predictive models across diverse cohorts, and clarifying the immune-regulatory mechanisms of necroptosis to strengthen its potential as a therapeutic target.

**Figure 12:**
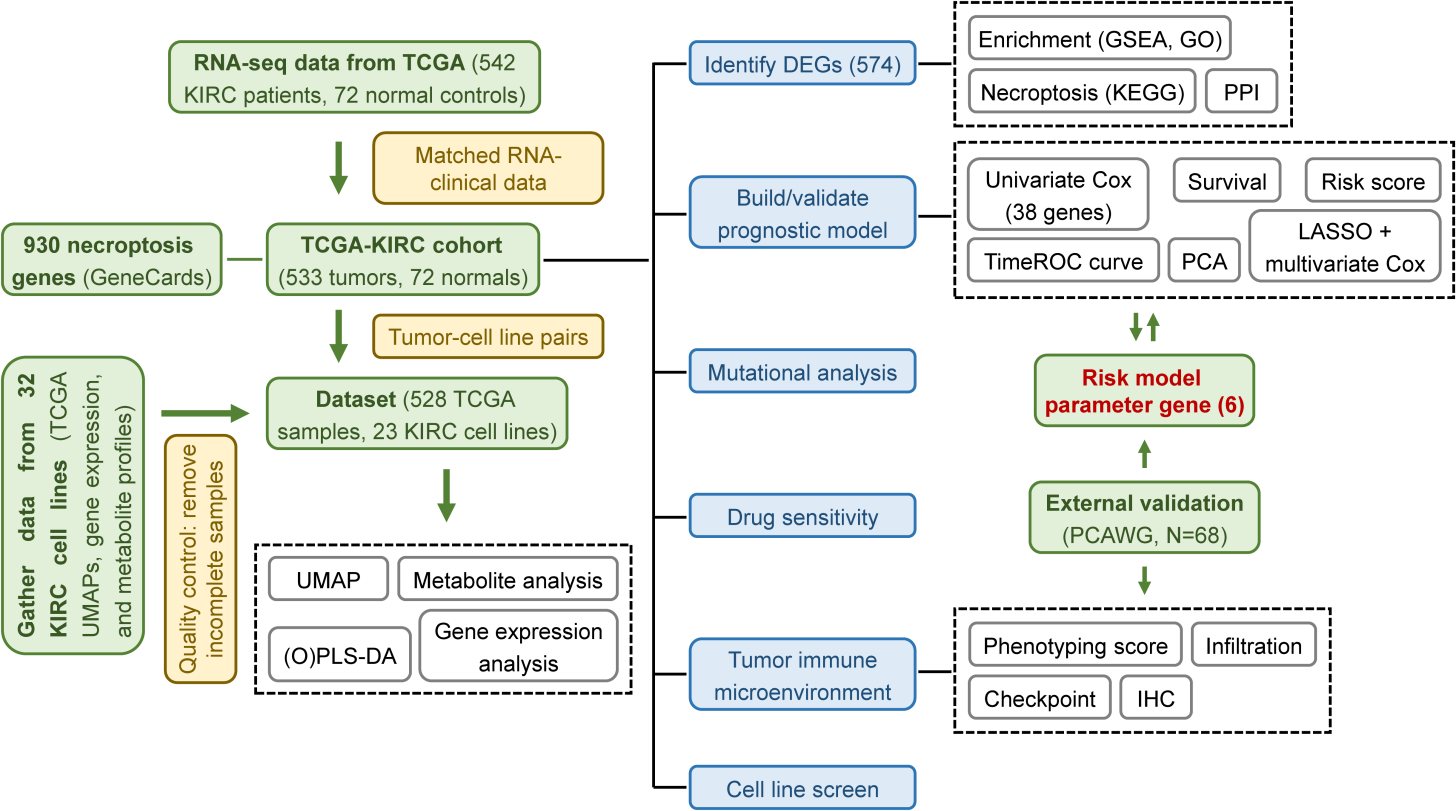
Schematic workflow of the study. Overview of the computational and experimental workflow used for model development, validation, and functional exploration.

Through our research (the overall flow diagram is shown in **Figure 12**), we identified nine core DEGs associated with KIRC—*RIPK1, RIPK3, MLKL, CASP8, ZBP1, TLR3, PYGL, TRPM7*, and *PGAM5*—all integral to the necroptotic signaling pathway. *CASP8* and *PGAM5* were identified as potential biomarkers among these, while previous studies have shown that *ZBP1, TLR3*, and *PYGL* contribute to tumor progression in KIRC (19–21). These genes are connected through intermediates such as TICAM1, TRADD, GSDMD, FADD, and CASP10. TICAM1 (TRIF) acts as an adaptor protein for TLR3, activating NF-κB and promoting interferon-β production in response to dsRNA, while contributing to necroptosis (20, 35). TRADD, involved in TNFR1 signaling, facilitates programmed cell death through its interaction with TRAF2 (36). GSDMD, a gasdermin family member, triggers cell membrane rupture upon activation, a hallmark of necroptosis (37). FADD, primarily associated with apoptosis, may influence necroptotic pathways, and CASP10, a cysteine protease, participates in programmed cell death but requires further characterization (38, 39). By applying a stringent differential gene screening threshold of p-value < 0.001—stricter than the commonly used p-value < 0.05 — and integrating KEGG pathway analyses, we ensured that selected genes were not only statistically significant but also biologically relevant to necroptosis. This comprehensive approach enhances the reliability of our findings.

For prognostic analysis, we developed a predictive model incorporating six necroptosis-associated genes: *IL4, CDC7, IGF2BP3, CASP9, TYRO3*, and *CPT2*. This model effectively predicts KIRC patient survival. Notably, *IL4* is a cytokine that promotes B-cell activity and antigen presentation (40); *CDC7* is an essential gene for the DNA replication process (41); *IGF2BP3* regulates mRNA stability and microRNA synthesis (42, 43); and *CASP9*, while central to apoptosis, promotes necroptosis when inhibited (44). Overexpression of *TYRO3*, a receptor tyrosine kinase, supports tumor proliferation and migration (45). Compared to traditional gene-selection approaches based on p-values, our method integrated HR changes rates for secondary screening, yielding a more refined and concise model. TimeROC analysis of the 6-gene signature demonstrated strong predictive performance, with 5-year survival AUC values of 0.77 in the training cohort and 0.89 in the independent/external validation cohort—surpassing the prognostic correlations of KIRC and other gene sets by at least 5.5% and 7.0%, respectively. Notably, within necroptosis-related gene sets, the independent/external validation cohort achieved an AUC improvement exceeding 17.1% (26, 46–48). In the mutational analysis, we analyzed TCGA-standardized VCF/MAF files using both maftools R package and iCoMut Beta tool on FireBrowse Platform. This approach successfully identified clinically significant driver mutations and yielded reliable prognostic biomarkers. However, as the analysis was based on exome sequencing data, it had certain detection limitations for low-frequency variants (VAF < 5%) and non-coding region alterations. To obtain more comprehensive genomic profiles, future studies may benefit from incorporating whole-genome sequencing or other advanced detection methods.

Tumor immune microenvironment analysis revealed greater immune cell infiltration and higher immunogenicity in high-risk groups, suggesting enhanced tumor microenvironment activity and better responsiveness to immunotherapy. This observation was validated in the PCAWG cohort and supported by existing literature (46). The immunosuppressive core in high-risk patients arised from MDSC-dominated metabolic inhibition and checkpoint amplification, rather than conventional Treg-mediated suppression. It created a paradoxical “high infiltration-low function” immune microenvironment. This microenvironment was characterized by elevated infiltration of functionally impaired CD8^+^ T cells alongside increased DCs and MDSCs, despite upregulation of immune pathways and heightened checkpoint expression (PD-1, CTLA4, CD72). Notably, Tregs were significantly reduced (p-value = 0.011), excluding their dominant role in immune suppression. CD8^+^ T cell exhaustion (mediated by PD-1/CTLA4) promoted MDSCs recruitment, while deletion at the 9p21.3 locus and *VHL* mutation-driven metabolic reprogramming (via HIF-1α/LDHA up-regulation) established a hypoxic, glycolytic niche that facilitated both MDSCs expansion and lactate-mediated T cell suppression (5–7, 49). Furthermore, tumor cell-derived PD-L1 reinforced this immunosuppressive cascade through positive feedback, ultimately explaining the coexistence of robust immune infiltration with profound functional impairment. Drug sensitivity analysis identified doxorubicin as a potential therapeutic option for KIRC.

IHC analysis also revealed the protein-level spatial distribution patterns of nine core genes and risk model parameters, thereby validating the prognostic model’s risk stratification. Through systematic screening of cell lines and target genes, our study identified BFTC909 and CAL54 as the most suitable model for subsequent experimental validation and revealed three novel target genes, including *CASP8*, *PGAM5* and *CPT2*, warranting further investigation.

In conclusion, our study establishes a strong connection between necroptosis and KIRC, uncovering significant differences in necroptosis-associated gene expression between normal and cancerous tissues. The six-gene prognostic model provides a valuable tool for predicting patient survival and underscores the potential of targeting necroptosis in KIRC treatment. We also highlight doxorubicin as a promising therapeutic agent and nominate the CAL54 through integrated RNA/protein analysis as the optimal cell line model along with three target genes for mechanistic studies. These findings contribute to a deeper understanding of KIRC’s molecular mechanisms, pave the way for innovative therapeutic strategies, and provide a data foundation for future exploration of liquid biopsy alternatives, validation of tissue-based scoring in treatment response, and development of simplified detection protocols.

## Data Availability

The original contributions presented in the study are included in the article/**Supplementary Material**. Further inquiries can be directed to the corresponding author.

## Supplementary material

Supplementary Figure 1 Rendering analysis of core DEGs in the KEGG-necroptotic signaling pathway. Colored genes represent core DEGs in the KEGG necroptosis pathway. Green: downregulated in tumors; red: upregulated. Color intensity indicates fold-change magnitude.

Supplementary Figure 2 Multifactorial subgroup survival analysis in the TCGA cohort.

Supplementary Figure 3 Genes Mutation diagram with clinical annotations for 533 TCGA samples.

Supplementary Figure 4 UMAP distribution of the TCGA cohort based on consensus clustering typing.

## References

[1] Jonasch E Walker CL Rathmell WK. Clear cell renal cell carcinoma ontogeny and mechanisms of lethality. Nat Rev Nephrology. (2020) 17:245–61. doi: 10.1038/s41581-020-00359-2, PMID: 33144689 PMC8172121

[2] Hsieh JJ Purdue MP Signoretti S Swanton C Albiges L Schmidinger M. Renal cell carcinoma. Nat Rev Dis Primers. (2017) 3:17009. doi: 10.1038/nrdp.2017.9, PMID: 28276433 PMC5936048

[3] Mao X Huang W Xue Q Zhang X. Prognostic impact and immunotherapeutic implications of NETosis-related prognostic model in clear cell renal cell carcinoma. J Cancer Res Clin Oncol. (2024) 150:278. doi: 10.1007/s00432-024-05761-y, PMID: 38801430 PMC11129999

[4] Qi X Li Q Che X Wang Q Wu G. The Uniqueness of Clear Cell Renal Cell Carcinoma: Summary of the Process and Abnormality of Glucose Metabolism and Lipid Metabolism in ccRCC. Front Oncol. (2021) 11:727778. doi: 10.3389/fonc.2021.727778, PMID: 34604067 PMC8479096

[5] Gnarra JR Tory K Weng Y Schmidt L Wei MH Li H. Mutations of the VHL tumour suppressor gene in renal carcinoma. Nat Genet. (1994) 7:85–90. doi: 10.1038/ng0594-85, PMID: 7915601

[6] Wang Y Suarez ER Kastrunes G de Campos NSP Abbas R Pivetta RS. Evolution of cell therapy for renal cell carcinoma. Mol Cancer. (2024) 23:8. doi: 10.1186/s12943-023-01911-x, PMID: 38195534 PMC10775455

[7] Haas NB Nathanson KL. Hereditary Kidney Cancer Syndromes. Adv Chronic Kidney Disease. (2014) 21:81–90. doi: 10.1053/j.ackd.2013.10.001, PMID: 24359990 PMC3872053

[8] Hakimi AA Pham CG Hsieh JJ. A clear picture of renal cell carcinoma. Nat Genet. (2013) 45:849–50. doi: 10.1038/ng.2708, PMID: 23892664

[9] Dalgliesh GL Furge K Greenman C Chen L Bignell G Butler A. Systematic sequencing of renal carcinoma reveals inactivation of histone modifying genes. Nature. (2010) 463:360–3. doi: 10.1038/nature08672, PMID: 20054297 PMC2820242

[10] Yan J Wan P Choksi S Liu ZG. Necroptosis and tumor progression. Trends Cancer. (2022) 8:21–7. doi: 10.1016/j.trecan.2021.09.003, PMID: 34627742 PMC8702466

[11] Yu X Yuan J Shi L Dai S Yue L Yan M. Necroptosis in bacterial infections. Front Immunol. (2024) 15:1394857. doi: 10.3389/fimmu.2024.1394857, PMID: 38933265 PMC11199740

[12] Kaiser WJ Sridharan H Huang C Mandal P Upton JW Gough PJ. Toll-like Receptor 3-mediated Necrosis via TRIF, RIP3, and MLKL. J Biol Chem. (2013) 288:31268–79. doi: 10.1074/jbc.M113.462341, PMID: 24019532 PMC3829437

[13] Maelfait J Liverpool L Bridgeman A Ragan KB Upton JW Rehwinkel J. Sensing of viral and endogenous RNA by ZBP1/DAI induces ecroptosis. EMBO J. (2017) 36:2529–43. doi: 10.15252/embj.201796476, PMID: 28716805 PMC5579359

[14] Kaczmarek A Vandenabeele P Krysko DV. Necroptosis: The Release of Damage-Associated Molecular Patterns and Its Physiological Relevance. Immunity. (2013) 38:209–23. doi: 10.1016/j.immuni.2013.02.003, PMID: 23438821

[15] Feng X Song Q Yu A Tang H Peng Z Wang X. Receptor-interacting protein kinase 3 is a predictor of survival and plays a tumor suppressive role in colorectal cancer. Neoplasma. (2015) 62:592–601. doi: 10.4149/neo_2015_071, PMID: 25997957

[16] Nicolè L Sanavia T Cappellesso R Maffeis V Akiba J Kawahara A. Necroptosis-driving genes RIPK1, RIPK3 and MLKL-pare associated with intratumoral CD3+and CD8+T cell density and predict prognosis in hepatocellular carcinoma. J ImmunoTherapy Cancer. (2022) 10:e004031. doi: 10.1136/jitc-2021-004031, PMID: 35264437 PMC8915343

[17] Gao W Wang X Zhou Y Wang X Yu Y. Autophagy, ferroptosis, pyroptosis, and necroptosis in tumor immunotherapy. Signal Transduction Targeted Ther. (2022) 7:196. doi: 10.1038/s41392-022-01046-3, PMID: 35725836 PMC9208265

[18] Colbert LE Fisher SB Hardy CW Hall WA Saka B Shelton JW. Pronecrotic mixed lineage kinase domain-like protein expression is a prognostic biomarker in patients with early-stage resected pancreatic adenocarcinoma. Cancer. (2013) 119:3148–55. doi: 10.1002/cncr.28144, PMID: 23720157 PMC4389890

[19] Zhang Y Wang T Mutailipu D Li Y Liang S Yi Q. ZBP1 as a prognostic biomarker correlated with cell proliferation in clear cell renal cell carcinoma. Heliyon. (2024) 10:e39267. doi: 10.1016/j.heliyon.2024.e39267, PMID: 39469683 PMC11513510

[20] Chen W Lin W Wu L Xu A Liu C Huang P. A Novel Prognostic Predictor of Immune Microenvironment and Therapeutic Response in Kidney Renal Clear Cell Carcinoma based on Necroptosis-related Gene Signature. Int J Med Sci. (2022) 19:377–92. doi: 10.7150/ijms.69060, PMID: 35165523 PMC8795799

[21] Li M Zhu G Liu Y Li X Zhou Y Li C. Integrated genomic and proteomic analyses identify PYGL as a novel experimental therapeutic target for clear cell renal cell carcinoma. Heliyon. (2024) 10:e28295. doi: 10.1016/j.heliyon.2024.e28295, PMID: 38545181 PMC10966709

[22] Kang YJ Bang BR Han KH Hong L Shim EJ Ma J. Regulation of NKT cell-mediated immune responses to tumours and liver inflammation by mitochondrial PGAM5-Drp1 signalling. Nat Commun. (2015) 6:8371. doi: 10.1038/ncomms9371, PMID: 26381214 PMC4576739

[23] Rucker AJ Chan FKM. Tumor-intrinsic and immune modulatory roles of receptor-interacting protein kinases. Trends Biochem Sci. (2022) 47:342–51. doi: 10.1016/j.tibs.2021.12.004, PMID: 34998669 PMC8917977

[24] Zhang M Liu YF Gao Y Zhao C Chen M Pan KH. Immune-pyroptosis-related genes predict the prognosis of kidney renal clear cell carcinoma. Trans Oncol. (2023) 34:101693. doi: 10.1016/j.tranon.2023.101693, PMID: 37315507 PMC10302855

[25] Hong Y Lin M Ou D Huang Z Shen P. A novel ferroptosis-related 12-gene signature predicts clinical prognosis and reveals immune relevancy in clear cell renal cell carcinoma. BMC Cancer. (2021) 21:831. doi: 10.1186/s12885-021-08559-0, PMID: 34281531 PMC8290606

[26] Li J Liu X Qi Y Liu Y Du E Zhang Z. A risk signature based on necroptotic-process-related genes predicts prognosis and immune therapy response in kidney cell carcinoma. Front Immunol. (2022) 13:922929. doi: 10.3389/fimmu.2022.922929, PMID: 36189275 PMC9524857

[27] Luo Y Zhang G. Identification of a Necroptosis-Related Prognostic Index and Associated Regulatory Axis in Kidney Renal Clear Cell Carcinoma. Int J Gen Med. (2022) 15:5407–23. doi: 10.2147/ijgm.S367173, PMID: 35685693 PMC9173730

[28] Wei K Zhang X Yang D. Identification and validation of prognostic and tumor microenvironment characteristics of necroptosis index and BIRC3 in clear cell renal cell carcinoma. PeerJ. (2023) 11:e16643. doi: 10.7717/peerj.16643, PMID: 38130918 PMC10734432

[29] Zhong C Xie T Chen L Zhong X Li X Cai X. Immune depletion of the methylated phenotype of colon cancer is closely related to resistance to immune checkpoint inhibitors. Front Immunol. (2022) 13:983636. doi: 10.3389/fimmu.2022.983636, PMID: 36159794 PMC9492852

[30] Wolff I May M Hoschke B Zigeuner R Cindolo L Hutterer G. Do we need new high-risk criteria for surgically treated renal cancer patients to improve the outcome of future clinical trials in the adjuvant setting? Results of a comprehensive analysis based on the multicenter CORONA database. Eur J Surg Oncol (EJSO). (2016) 42:744–50. doi: 10.1016/j.ejso.2016.01.009, PMID: 26899942

[31] Naik P Dudipala H Chen YW Rose B Bagrodia A McKay RR. The incidence, pathogenesis, and management of non-clear cell renal cell carcinoma. Ther Adv Urology. (2024) 16:17562872241232578. doi: 10.1177/17562872241232578, PMID: 38434237 PMC10906063

[32] Sun L Wang H Wang Z He S Chen S Liao D. Mixed Lineage Kinase Domain-like Protein Mediates Necrosis Signaling Downstream of RIP3 Kinase. Cell. (2012) 148:213–27. doi: 10.1016/j.cell.2011.11.031, PMID: 22265413

[33] Zhang DW Shao J Lin J Zhang N Lu BJ Lin SC. RIP3, an Energy Metabolism Regulator That Switches TNF-Induced Cell Death from Apoptosis to Necrosis. Science. (2009) 325:332–6. doi: 10.1126/science.1172308, PMID: 19498109

[34] Orozco SL Daniels BP Yatim N Messmer MN Quarato G Harris H. RIPK3 Activation Leads to Cytokine Synthesis that Continues after Loss of Cell Membrane Integrity. Cell Rep. (2019) 28:2275–2287.e2275. doi: 10.1016/j.celrep.2019.07.077, PMID: 31461645 PMC6857709

[35] Luo L Lucas RM Liu L Stow JL. Signalling, sorting and scaffolding adaptors for Toll-like receptors. J Cell Science. (2020) 133:jcs239194. doi: 10.1242/jcs.239194, PMID: 31889021

[36] Li Z Yuan W Lin Z. Functional roles in cell signaling of adaptor protein TRADD from a structural perspective. Comput Struct Biotechnol J. (2020) 18:2867–76. doi: 10.1016/j.csbj.2020.10.008, PMID: 33163147 PMC7593343

[37] Frank D Vince JE. Pyroptosis versus necroptosis: similarities, differences, and crosstalk. Cell Death Differentiation. (2018) 26:99–114. doi: 10.1038/s41418-018-0212-6, PMID: 30341423 PMC6294779

[38] Lee EW Seo JH Jeong MH Lee SS Song JW. The roles of FADD in extrinsic apoptosis and necroptosis. BMB Rep. (2012) 45:496–508. doi: 10.5483/BMBRep.2012.45.9.186, PMID: 23010170

[39] Matas Pérez E Valdivieso Shephard JL Bravo García Morato M Robles Marhuenda Á Martinez-Ojinaga Nodal E Prieto Bozano G. Variants in CASP10, a diagnostic challenge: Single center experience and review of the literature. Clin Immunol. (2021) 230:108812. doi: 10.1016/j.clim.2021.108812, PMID: 34329798

[40] Bankaitis KV Fingleton B. Targeting IL4/IL4R for the treatment of epithelial cancer metastasis. Clin Exp Metastasis. (2015) 32:847–56. doi: 10.1007/s10585-015-9747-9, PMID: 26385103 PMC4651701

[41] Gonzalez GC Prado F. Novel insights into the roles of Cdc7 in response to replication stress. FEBS J. (2023) 290:3076–88. doi: 10.1111/febs.16456, PMID: 35398961

[42] Liu X Chen J Chen W Xu Y Shen Y Xu X. Targeting IGF2BP3 in Cancer. Int J Mol Sci. (2023) 24:9423. doi: 10.3390/ijms24119423, PMID: 37298373 PMC10253605

[43] Li GX Chen L Hsiao Y Mannan R Zhang Y Luo J. Comprehensive proteogenomic characterization of rare kidney tumors. Cell Rep Med. (2024) 5:101547. doi: 10.1016/j.xcrm.2024.101547, PMID: 38703764 PMC11148773

[44] Xue Q Kang R Klionsky DJ Tang D Liu J Chen X. Copper metabolism in cell death and autophagy. Autophagy. (2023) 19:2175–95. doi: 10.1080/15548627.2023.2200554, PMID: 37055935 PMC10351475

[45] Hsu PL Jou J Tsai SJ. TYRO3: A potential therapeutic target in cancer. Exp Biol Med. (2019) 244:83–99. doi: 10.1177/1535370219828195, PMID: 30714403 PMC6405828

[46] Shi N Chen S Wang D Wu T Zhang N Chen M. MDK promotes M2 macrophage polarization to remodel the tumour microenvironment in clear cell renal cell carcinoma. Sci Rep. (2024) 14:18254. doi: 10.1038/s41598-024-69183-z, PMID: 39107475 PMC11303797

[47] Fu L Bao J Li J Li Q Lin H Zhou Y. Crosstalk of necroptosis and pyroptosis defines tumor microenvironment characterization and predicts prognosis in clear cell renal carcinoma. Front Immunol. (2022) 13:1021935. doi: 10.3389/fimmu.2022.1021935, PMID: 36248876 PMC9561249

[48] Wang H Liu Z Du Y Cheng X Gao S Gao Y. TPD52L2 as a potential prognostic and immunotherapy biomarker in clear cell renal cell carcinoma. Front Oncol. (2023) 13:1210910. doi: 10.3389/fonc.2023.1210910, PMID: 38074636 PMC10701739

[49] Braun DA Hou Y Bakouny Z Ficial M Sant' Angelo M Forman J. Interplay of somatic alterations and immune infiltration modulates response to PD-1 blockade in advanced clear cell renal cell carcinoma. Nat Med. (2020) 26:909–18. doi: 10.1038/s41591-020-0839-y, PMID: 32472114 PMC7499153

