# Supplementary figures and images for: Identification of necroptosis-associated mRNA biomarkers in kidney clear cell carcinoma
Source: Front Immunol. 2025 Sep 3;16:1545486. doi: 10.3389/fimmu.2025.1545486 (PMC12440986; doi:10.3389/fimmu.2025.1545486)

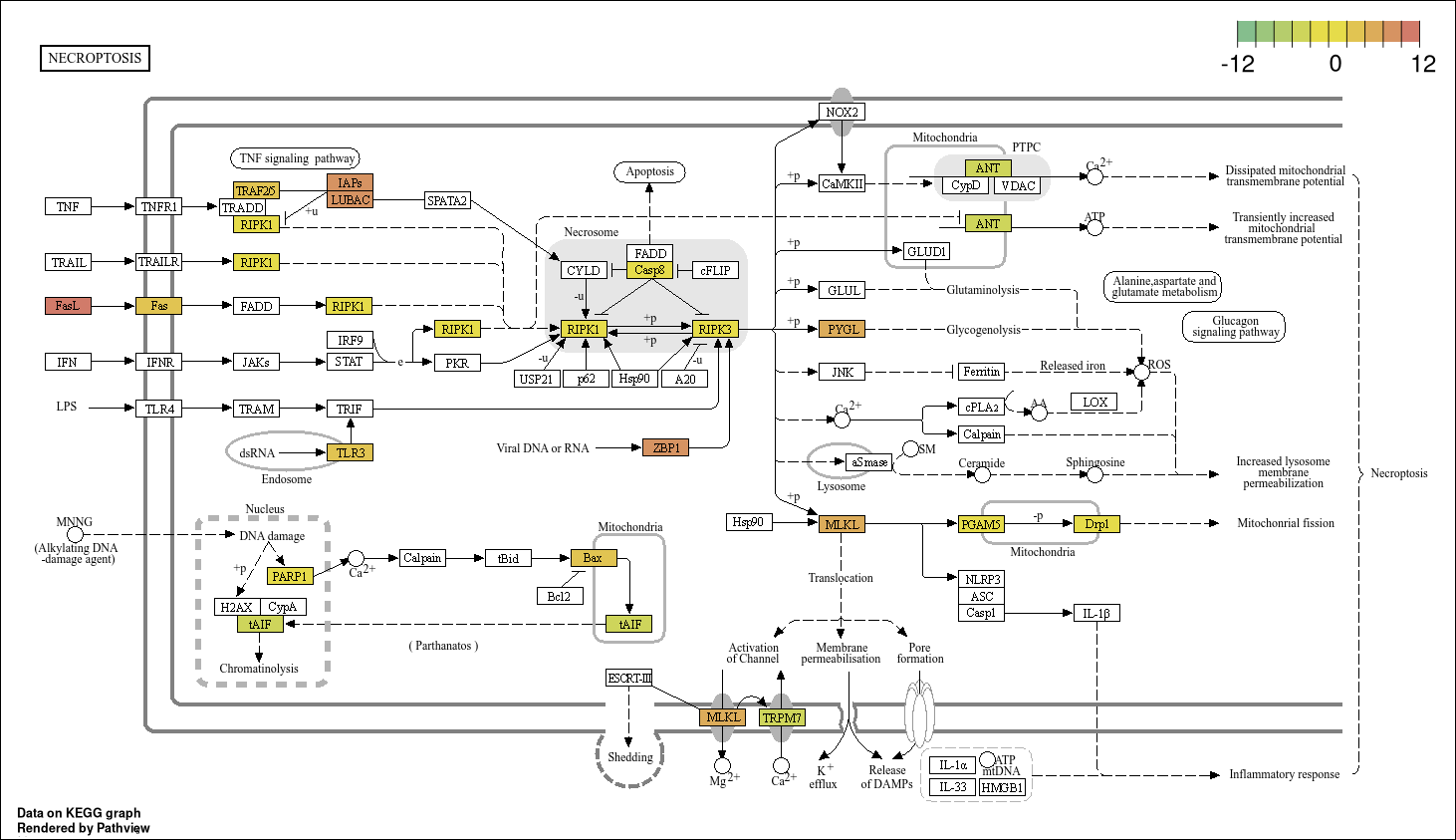

Supplement: Supplementary Figure 1 — Rendering analysis of core DEGs in the KEGG-necroptotic signaling pathway. Colored genes represent core DEGs in the KEGG necroptosis pathway. Green: downregulated in tumors; red: upregulated. Color intensity indicates fold-change magnitude. [file Image1.tif]

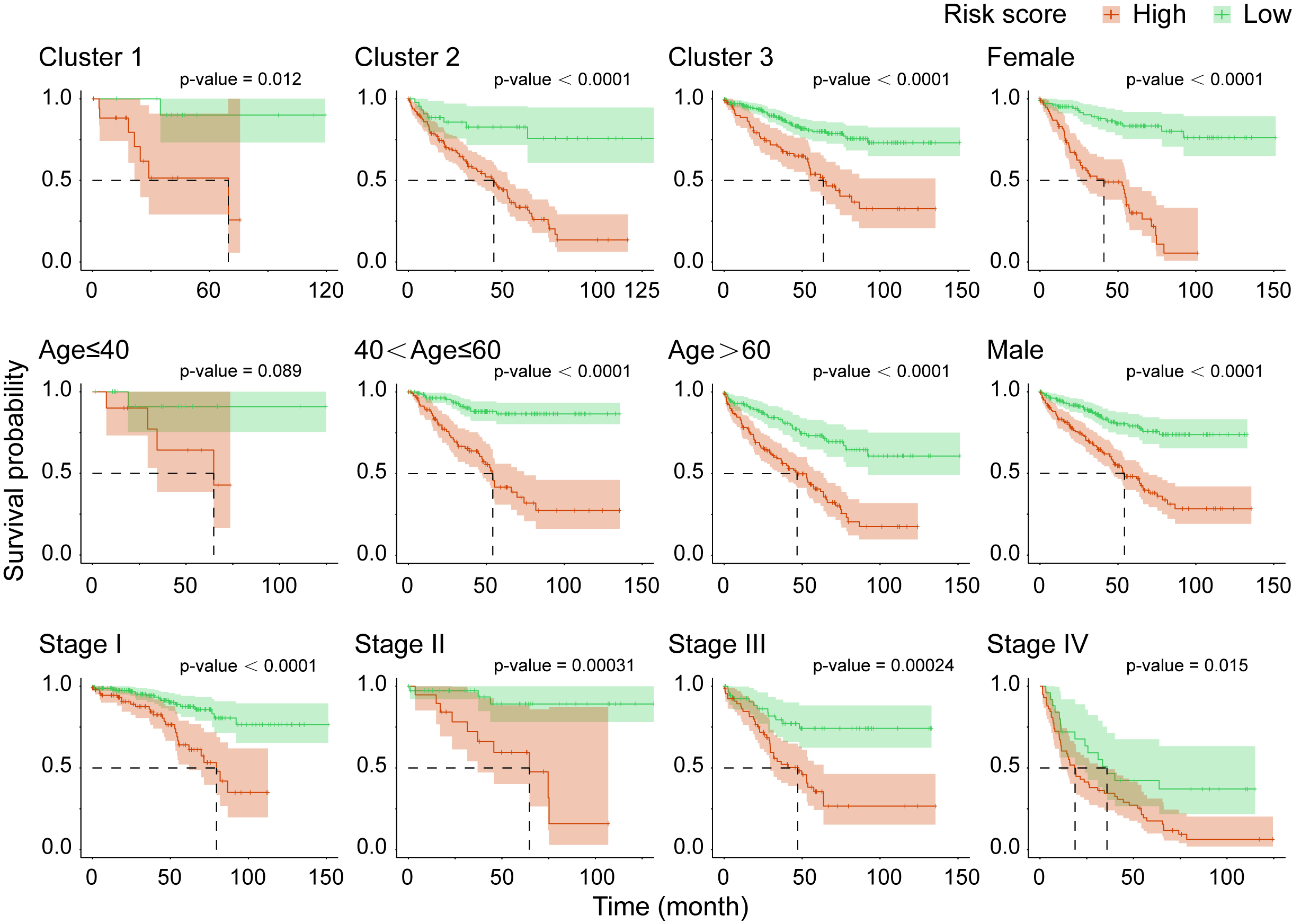

Supplement: Supplementary Figure 2 — Multifactorial subgroup survival analysis in the TCGA cohort. [file Image2.tif]

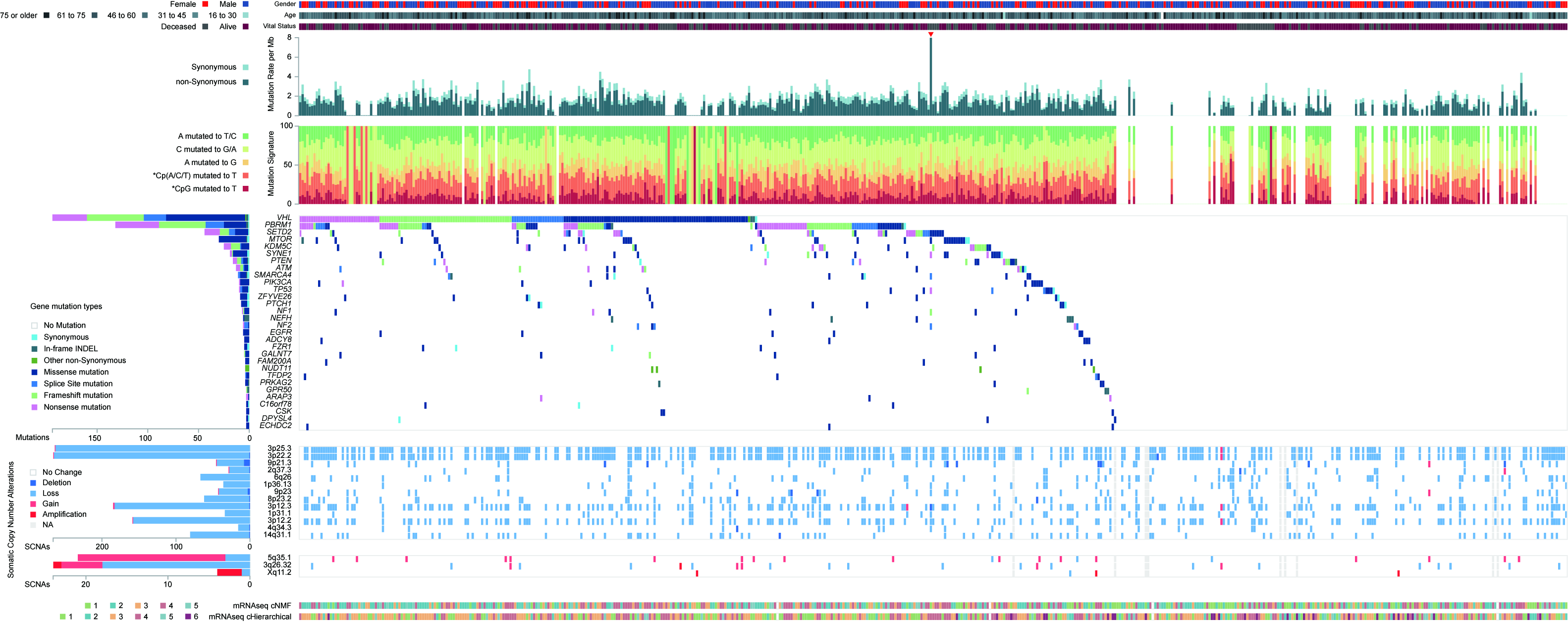

Supplement: Supplementary Figure 3 — Genes Mutation diagram with clinical annotations for 533 TCGA samples. [file Image3.tif]

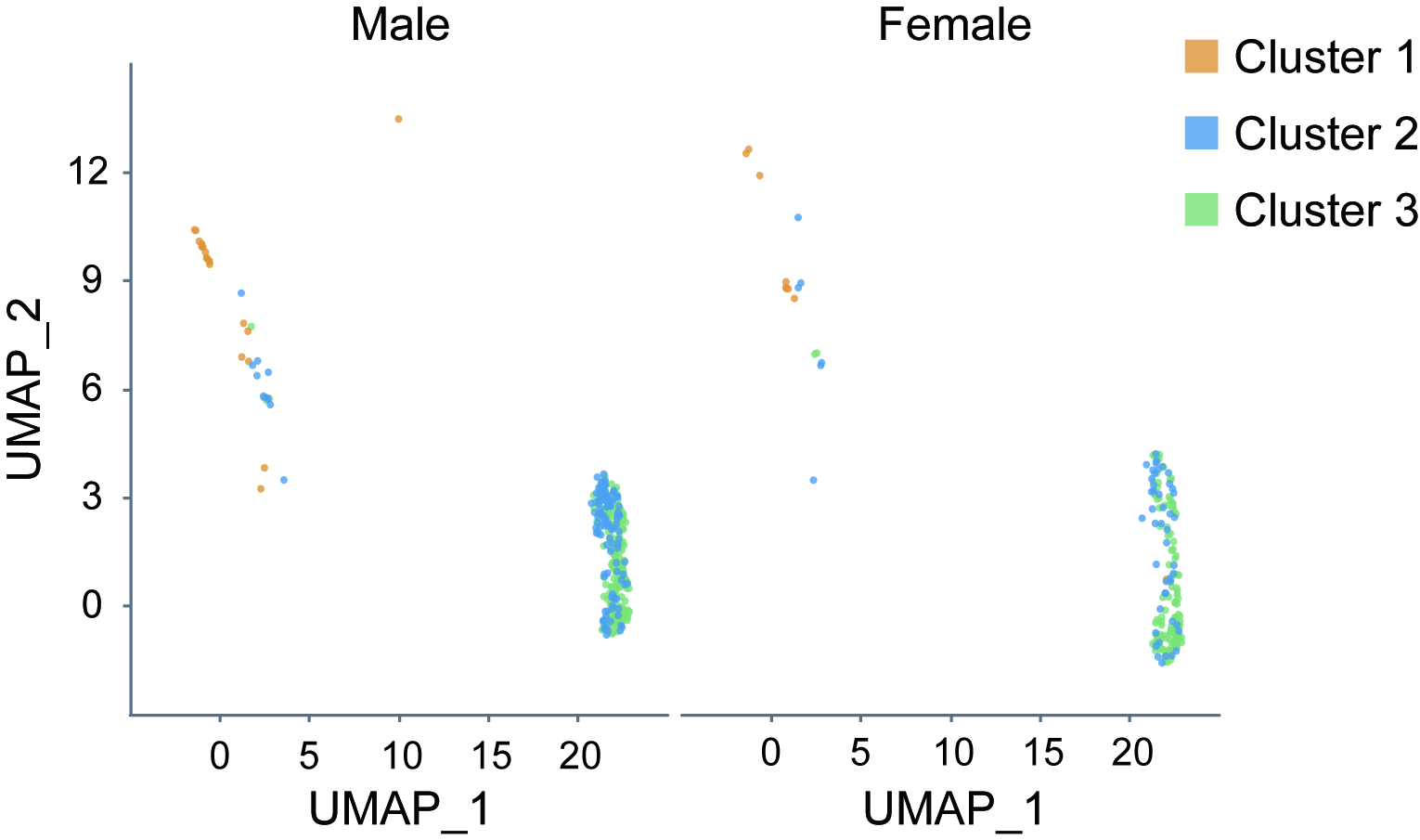

Supplement: Supplementary Figure 4 — UMAP distribution of the TCGA cohort based on consensus clustering typing. [file Image4.tif]
