# Supplementary material for: Identification of necroptosis-associated mRNA biomarkers in kidney clear cell carcinoma
Source: Front Immunol. 2025 Sep 3;16:1545486. doi: 10.3389/fimmu.2025.1545486 (PMC12440986; doi:10.3389/fimmu.2025.1545486)
Supplement: Supplementary file 5 [file Table1.docx]

**Supplementary Table 1 Univariate Cox analysis results**

| **Gene ID** | **HR** | **HR.95 L** | **HR.95H** | **p-value** | **δHR (%)** |
| --- | --- | --- | --- | --- | --- |
| *IL4* | 1.1305 | 1.0943 | 1.1679 | 1.44E-13 | 1.30E-01 |
| *RNF31* | 1.0106 | 1.0059 | 1.0152 | 7.06E-06 | 1.06E-02 |
| *RPS10* | 1.0084 | 1.0059 | 1.0110 | 4.19E-11 | 8.44E-03 |
| *AHRR* | 1.0074 | 1.0006 | 1.0143 | 3.35E-02 | 7.44E-03 |
| *AURKC* | 1.0056 | 1.0030 | 1.0082 | 2.70E-05 | 5.62E-03 |
| *IFNG* | 1.0026 | 1.0017 | 1.0034 | 8.62E-10 | 2.56E-03 |
| *ZSCAN20* | 0.9977 | 0.9961 | 0.9993 | 4.43E-03 | -2.32E-03 |
| *TLE6* | 1.0023 | 1.0010 | 1.0035 | 2.69E-04 | 2.25E-03 |
| *CDC7* | 1.0019 | 1.0014 | 1.0025 | 1.05E-11 | 1.95E-03 |
| *TNFRSF8* | 1.0017 | 1.0009 | 1.0026 | 5.80E-05 | 1.72E-03 |
| *TP63* | 1.0017 | 1.0002 | 1.0033 | 2.84E-02 | 1.71E-03 |
| *RIPK3* | 1.0015 | 1.0002 | 1.0029 | 2.45E-02 | 1.53E-03 |
| *BUB1B* | 1.0014 | 1.0009 | 1.0018 | 6.00E-11 | 1.35E-03 |
| *STAT4* | 1.0013 | 1.0005 | 1.0022 | 2.17E-03 | 1.31E-03 |
| *MEFV* | 1.0013 | 1.0004 | 1.0022 | 6.93E-03 | 1.29E-03 |
| *EZH2* | 1.0012 | 1.0008 | 1.0016 | 3.02E-08 | 1.21E-03 |
| *ZBP1* | 1.0011 | 1.0006 | 1.0016 | 2.85E-06 | 1.10E-03 |
| *ESR2* | 1.0010 | 1.0003 | 1.0017 | 4.05E-03 | 1.03E-03 |
| *CPT2* | 0.9991 | 0.9987 | 0.9994 | 1.20E-07 | -9.43E-04 |
| *TNIP3* | 1.0009 | 1.0002 | 1.0015 | 6.41E-03 | 8.82E-04 |
| *SIRT6* | 1.0009 | 1.0004 | 1.0013 | 1.65E-04 | 8.61E-04 |
| *BAG2* | 1.0008 | 1.0005 | 1.0011 | 6.52E-07 | 8.10E-04 |
| *AURKA* | 1.0008 | 1.0006 | 1.0010 | 7.63E-13 | 7.76E-04 |
| *FAM83D* | 1.0007 | 1.0004 | 1.0010 | 1.40E-06 | 7.45E-04 |
| *PABPN1* | 1.0007 | 1.0005 | 1.0010 | 5.23E-08 | 7.43E-04 |
| *CRTAM* | 1.0007 | 1.0001 | 1.0014 | 2.07E-02 | 7.41E-04 |
| *ZNF7* | 1.0007 | 1.0003 | 1.0011 | 7.60E-04 | 7.17E-04 |
| *KIF11* | 1.0007 | 1.0005 | 1.0009 | 2.32E-11 | 7.15E-04 |
| *FASLG* | 1.0007 | 1.0001 | 1.0013 | 2.23E-02 | 7.07E-04 |
| *IGF2BP3* | 1.0007 | 1.0005 | 1.0009 | 4.30E-11 | 6.86E-04 |
| *TYRO3* | 0.9994 | 0.9990 | 0.9997 | 7.42E-04 | 6.42E-04 |
| *TNFRSF10A* | 0.9994 | 0.9990 | 0.9998 | 4.62E-03 | -5.99E-04 |
| *USP21* | 1.0006 | 1.0003 | 1.0009 | 1.32E-04 | 5.86E-04 |
| *BID* | 1.0006 | 1.0004 | 1.0008 | 2.56E-09 | 5.69E-04 |
| *CASP9* | 1.0006 | 1.0002 | 1.0009 | 2.30E-03 | 5.60E-04 |
| *FAP* | 1.0006 | 1.0003 | 1.0008 | 1.41E-04 | 5.52E-04 |
| *STUB1* | 0.9995 | 0.9991 | 0.9998 | 8.52E-04 | -5.37E-04 |
| *SNRPF* | 1.0005 | 1.0003 | 1.0007 | 2.46E-10 | 5.00E-04 |
