# Supplementary material for: Identification of necroptosis-associated mRNA biomarkers in kidney clear cell carcinoma
Source: Front Immunol. 2025 Sep 3;16:1545486. doi: 10.3389/fimmu.2025.1545486 (PMC12440986; doi:10.3389/fimmu.2025.1545486)
Supplement: Supplementary file 6 [file Table2.docx]

**Supplementary Table 2 Results of multivariate Cox analysis**

| **Gene ID** | **Coef** | **HR** | **HR.95 L** | **HR.95H** | **p-value** |
| --- | --- | --- | --- | --- | --- |
| *IL4* | 0.8940 | 2.4448 | 1.3065 | 4.5751 | 0.0052 |
| *CDC7* | 1.5176 | 4.5612 | 1.4920 | 13.9442 | 0.0078 |
| *IGF2BP3* | 0.4019 | 1.4946 | 1.0951 | 2.0400 | 0.0113 |
| *CASP9* | 1.8612 | 6.4312 | 1.4640 | 28.2512 | 0.0137 |
| *TYRO3* | -0.8737 | 0.4173 | 0.1846 | 0.9439 | 0.0359 |
| *CPT2* | -1.0633 | 0.3453 | 0.1268 | 0.9407 | 0.0376 |
| *BID* | 1.4246 | 4.1561 | 0.9215 | 18.7440 | 0.0638 |
| *AURKC* | -0.8489 | 0.4279 | 0.1693 | 1.0815 | 0.0727 |
| *CRTAM* | -0.9084 | 0.4032 | 0.1410 | 1.1526 | 0.0901 |
| *SNRPF* | -1.2508 | 0.2863 | 0.0591 | 1.3865 | 0.1202 |
| *ZSCAN20* | -1.0225 | 0.3597 | 0.0958 | 1.3506 | 0.1298 |
| *STAT4* | -0.8471 | 0.4287 | 0.1329 | 1.3820 | 0.1561 |
| *RIPK3* | 0.7019 | 2.0175 | 0.7331 | 5.5525 | 0.1742 |
| *TNFRSF10A* | -0.5317 | 0.5876 | 0.2506 | 1.3777 | 0.2213 |
| *ZNF7* | 1.2002 | 3.3207 | 0.4777 | 23.0822 | 0.2250 |
| *FAP* | -0.3192 | 0.7268 | 0.3971 | 1.3301 | 0.3007 |
| *USP21* | -0.9055 | 0.4043 | 0.0687 | 2.3805 | 0.3168 |
| *TLE6* | -0.2865 | 0.7509 | 0.4174 | 1.3507 | 0.3388 |
| *ZBP1* | 0.4505 | 1.5691 | 0.6212 | 3.9635 | 0.3407 |
| *TP63* | -0.2477 | 0.7806 | 0.4620 | 1.3189 | 0.3547 |
| *TNFRSF8* | 0.3729 | 1.4520 | 0.6344 | 3.3232 | 0.3774 |
| *EZH2* | -0.7036 | 0.4948 | 0.0997 | 2.4561 | 0.3894 |
| *IFNG* | 0.2516 | 1.2860 | 0.6566 | 2.5189 | 0.4633 |
| *FASLG* | 0.3324 | 1.3943 | 0.4959 | 3.9201 | 0.5285 |
| *BAG2* | 0.2056 | 1.2283 | 0.6428 | 2.3470 | 0.5337 |
| *PABPN1* | 0.3489 | 1.4175 | 0.4316 | 4.6561 | 0.5653 |
| *STUB1* | -0.3295 | 0.7193 | 0.2316 | 2.2336 | 0.5687 |
| *ESR2* | -0.1499 | 0.8608 | 0.4894 | 1.5138 | 0.6027 |
| *FAM83D* | -0.1987 | 0.8198 | 0.3556 | 1.8901 | 0.6411 |
| *AHRR* | -0.1061 | 0.8994 | 0.5741 | 1.4089 | 0.6433 |
| *TNIP3* | 0.0816 | 1.0850 | 0.6160 | 1.9112 | 0.7775 |
| *SIRT6* | 0.1829 | 1.2007 | 0.2217 | 6.5013 | 0.8320 |
| *MEFV* | -0.0627 | 0.9392 | 0.5092 | 1.7321 | 0.8408 |
| *KIF11* | 0.1770 | 1.1937 | 0.2064 | 6.9032 | 0.8433 |
| *BUB1B* | 0.1397 | 1.1499 | 0.2828 | 4.6755 | 0.8453 |
| *AURKA* | 0.0574 | 1.0591 | 0.2730 | 4.1092 | 0.9338 |
| *RNF31* | -0.0476 | 0.9535 | 0.3078 | 2.9538 | 0.9342 |
| *RPS10* | 0.0249 | 1.0252 | 0.3516 | 2.9893 | 0.9636 |
