# Supplementary material for: Identification of necroptosis-associated mRNA biomarkers in kidney clear cell carcinoma
Source: Front Immunol. 2025 Sep 3;16:1545486. doi: 10.3389/fimmu.2025.1545486 (PMC12440986; doi:10.3389/fimmu.2025.1545486)
Supplement: Supplementary file 7 [file Table3.docx]

**Supplementary Table 3 VIP values of 23 cell lines based on (O)PLS-DA**

| **Cell lines** | **DepMap ID** | **Cluster-VIP** | **Risk-VIP** | **Average-VIP** |
| --- | --- | --- | --- | --- |
| 769P | ACH-000411 | 0.3453 | 0.9629 | 0.6541 |
| 786O | ACH-000649 | 0.8203 | 1.1187 | 0.9695 |
| A498 | ACH-000555 | 0.5776 | 1.0564 | 0.8170 |
| A704 | ACH-000429 | 3.3033 | 1.1168 | 2.2101 |
| ACHN | ACH-000046 | 0.8642 | 0.8811 | 0.8727 |
| BFTC909 | ACH-000792 | 1.6551 | 1.2100 | 1.4326 |
| CAKI1 | ACH-000433 | 0.8910 | 0.9563 | 0.9237 |
| CAKI2 | ACH-000234 | 0.7962 | 0.9925 | 0.8944 |
| CAL54 | ACH-000457 | 1.3201 | 0.9087 | 1.1144 |
| KMRC1 | ACH-000684 | 0.4621 | 1.0393 | 0.7507 |
| KMRC2 | ACH-000709 | 0.3733 | 0.9442 | 0.6588 |
| KMRC20 | ACH-000250 | 0.6713 | 1.0547 | 0.8630 |
| KMRC3 | ACH-000313 | 0.4586 | 0.6862 | 0.5724 |
| OSRC2 | ACH-000159 | 0.3424 | 1.1181 | 0.7303 |
| RCC10RGB | ACH-000189 | 0.2908 | 1.0427 | 0.6668 |
| SNU1272 | ACH-000513 | 0.2783 | 0.8975 | 0.5879 |
| SNU349 | ACH-000907 | 0.0672 | 0.9389 | 0.5031 |
| TUHR10TKB | ACH-000459 | 0.3266 | 0.9557 | 0.6412 |
| TUHR14TKB | ACH-000317 | 0.3098 | 1.0251 | 0.6675 |
| TUHR4TKB | ACH-000495 | 0.1301 | 1.0293 | 0.5797 |
| UO31 | ACH-000428 | 1.6238 | 1.0257 | 1.3248 |
| VMRCRCW | ACH-000484 | 0.2644 | 0.8318 | 0.5481 |
| VMRCRCZ | ACH-000171 | 0.2860 | 1.0703 | 0.6782 |
